# Being treated fairly in groups is important, but not sufficient: The role of distinctive treatment in groups, and its implications for mental health

**DOI:** 10.1371/journal.pone.0251871

**Published:** 2021-05-14

**Authors:** Christopher T. Begeny, Yuen J. Huo, Heather J. Smith, Michelle K. Ryan

**Affiliations:** 1 Department of Psychology, University of Exeter, Exeter, United Kingdom; 2 Department of Psychology, University of California, Los Angeles, Los Angeles, California, United States of America; 3 Department of Psychology, Sonoma State University, Rohnert Park, California, United States of America; 4 Faculty of Economics and Business, University of Groningen, Groningen, The Netherlands; Montclair State University, UNITED STATES

## Abstract

Organizations and other groups often recognize the importance of members treating each other in a fair (dignified, unbiased) manner. This type of treatment is key to fostering individuals’ sense of belonging in the group. However, while a sense of belonging is important, individuals also need to be shown that they have some distinct value to the group–enabling them to not only “fit in” but also “stand out.” Building from research on fair treatment, we explicate another form, *distinctive treatment*, whereby others show interest and appreciation for an individual’s more distinguishing, group-relevant qualities. In six studies using multiple methods (e.g., experimental, longitudinal) and in multiple types of groups (work organizations, student communities, racial/ethnic minority groups), we show that fair and distinctive treatment play fundamentally different roles–shaping individuals’ perceived belonging versus intragroup standing, respectively–and with downstream benefits for mental health (less anxiety, fewer depressive symptoms). Overall, this illustrates that promoting fair treatment in groups is important, but not sufficient. Experiencing distinctive treatment is also key. Each type of treatment provides unique social evaluative information that fosters a healthy sense of self. This research further indicates that distinctive treatment may be a vital yet overlooked element to promoting diversity and inclusion in groups, as it provides a path for recognizing and appreciating, and thus encouraging, a diversity of ideas, insights, knowledge and skills that individuals bring to the group.

## Introduction

Organizations often emphasize the importance of employees treating one another in ways that are fair (unbiased, and dignified). Educational institutions put a similar emphasis in how students should treat their peers. In part, these efforts reflect an awareness that this type of treatment is vital to promoting individuals’ sense of inclusion and belonging in groups [1–3]. Yet, while organizations and other groups should certainly encourage members to treat each other fairly, focusing on this type of treatment alone may be too narrow. This is because individuals want to not only “fit in” (to feel a sense of belonging) but also “stand out” within a group–to feel that they have some distinct value and worth they bring to the group and their fellow members. Both are vital for promoting individuals’ health and well-being [2] and, as we contend here, hinge on experiencing two distinct forms of group-based treatment.

In the current research, we build on insights from the procedural justice literature [4–8] by delineating a separate form of treatment, *distinctive treatment*–whereby others convey interest and appreciation for an individual’s particular group-relevant qualities (e.g., the skills, perspectives or knowledge they possess, and that can be an asset to the group). Across six studies, we examine the importance of experiencing distinctive treatment in groups, in tandem with the oft-studied fair treatment [4, 5, 7]. We posit that, together, these two forms of treatment enable individuals to not only “fit in” (to feel a sense of belonging, via fair treatment) but also “stand out” within a group (to feel a sense of distinct value and worth to the group, via distinctive treatment). We test their importance within a variety of groups: work organizations, student communities, and racial/ethnic minority groups. Notably, these represent some of the most common and meaningful groups that individuals engage with in everyday life. Thus, with this research we aim to shed new light on individuals’ everyday interactions in a variety of important groups–testing if among fellow group members, individuals are not only attuned to whether others treat them in a fair manner, but also whether others convey interest and appreciation for the skills and insights they bring to the group.

### Why group interactions matter to individuals

Within a self-relevant group, individuals are motivated to understand what the group thinks of them [9], which they generally discern based on how other members treat them during everyday interactions. During these interactions, group members provide valuable cues, or social evaluative information, that signal to individuals what the group thinks of them. This in turn shapes how those individuals see themselves within the group–that is, their group-based appraisals of the self. This is in fact a key observation in relational group models: that how individuals come to see themselves within a group is based on how other members treat them [4, 6, 10]. In particular, research has shown that experiencing *fair treatment* in a group–treatment that is unbiased, considerate, and dignified [7]–affects individuals’ group-based appraisals of the self (evinced via experimental and non-experimental methods; for reviews, see [5, 8]).

Importantly however, this body of work is varied in how it conceptualizes that aspect of an individual’s self that is centrally affected by fair treatment. Often, the focus has been on how fair treatment shapes individuals’ sense of ‘respect’ within the group. However, notions of respect have been conceptualized and operationalized variably, including at times being more akin to individuals’ sense of belonging within the group and at other times more akin to their sense of intragroup standing [5]. Several lines of work now indicate, however, that belonging and standing are markedly distinct aspects of an individual’s group-based self-appraisal, which can be thought of as fitting under the broader conceptual umbrella of ‘respect.’ This conceptual distinction is articulated in the dual pathway model of respect [5] (similarly see [1, 11, 12]). While intragroup *belonging* reflects a sense of being included and well-liked in the group (a sense of “fitting in”), intragroup *standing* reflects a sense of being looked up to, admired, and highly regarded among other members (a sense of “standing out”). Evidence has also shown that each positively contributes to health and well-being [2, 5], indicating that individuals need to develop *both* a sense of belonging and intragroup standing to thrive. Yet because of the conceptual variability in past work, it remains unclear whether experiencing fair treatment sufficiently provides both (equally), or if it more reliably provides one but does less to foster the other.

### Fair treatment conveys belonging

To discern whether fair treatment does more to shape individuals’ sense of belonging or their standing in groups, it is important to consider that past theorizing has generally regarded fair treatment as a basic right or entitlement due to all group members [7] (also [13, 14]). Thus, all group members can and should be treated in a generally fair (unbiased, dignified) manner. In support of this notion, when members of a group had to decide how they would treat individuals who either adhered to group norms or violated them, evidence showed that while members treated norm violators differently in certain respects (e.g., sharing fewer resources), in terms of treating them with dignity (fair treatment), members indicated that violators and non-violators should be treated alike [15] (similarly, see [16]). These findings support the idea that fair treatment is something that all members of a group are seen as equally entitled to (even those who violate group norms). Moreover, because it is seen as a basic entitlement, group members are likely to *expect* to be treated in a generally fair way.

Given that fair treatment is a general expectation across group members, this also likely shapes the type of information individuals can reliably derive about themselves when it is received. Specifically, if the presumed basis for distributing this type of treatment is not individuals’ particular standing in the group but instead whether they are considered full-fledged members, it follows that receiving it should not suggest much about their standing. Instead, by virtue of its nature as a basic entitlement to group members, fair treatment should mainly provide information that shapes individuals’ sense of belonging. Tyler and Blader [17] similarly suggest that when individuals are treated in ways that meet basic expectations within a group (treatment that aligns to “the group’s typical standards;” pg. 814), they develop a sense of belonging. Huo [15] further suggests a *lack* of fair treatment signals that individuals are not regarded as belonging to that group.

### Distinctive treatment and perceived standing

We contend that fair treatment is vital to promoting a sense of belonging in groups, but may not so readily provide information that helps individuals discern their intragroup standing. This raises the question of how individuals may then focally discern their standing in groups. Fortunately, previous work on group dynamics provides some insight into how other members’ treatment of an individual might ultimately shape that individual’s own sense of their standing. In particular, research on the characteristics of individuals who are admired by fellow group members shows that highly admired individuals tend to: (i) possess skills or knowledge that are valuable to the group, and/or (ii) behave in ways that serve and promote the group’s goals. Another critically important element characterizing those who are highly admired is that: (iii) others in the group are willing to *seek them out* for their advice, knowledge, and skills [18]. This third element is particularly important because it suggests a concrete, behavioral mechanism through which information about one’s standing in the group is communicated. It suggests how the actions of other group members–the act of going to an individual to seek out their guidance–may shape that individual’s perceptions of their own intragroup standing.

Together, this suggests individuals’ perceived standing in a group may be shaped by instances where other members seek out their guidance or call upon them to utilize a group-relevant skill, perspective, or base of knowledge they possess. Such instances may represent one of the key elements embedded in group-based interactions that effectively convey recognition of an individual’s particular group-relevant qualities, and thus shape the individual’s own appraisal of their standing in the group.

In conjunction with past work on fair treatment, these insights suggest that when interacting with fellow group members individuals are not only attuned to whether others convey fairness toward them–cues that shape their sense of belonging–but also whether others convey interest and appreciation for their particular skills, ideas, or group-relevant knowledge–cues that shape their sense of standing in the group. Therefore, we posit that individuals’ attention to these two sets of cues reflect attention to two distinct forms of group-based treatment. Each contains unique social evaluative information that individuals rely on to guide their sense of belonging and standing within groups, respectively.

#### Conceptualizing distinctive treatment

Building from these insights, we propose that individuals discern their standing in groups based on their experiences with a form of group-based treatment called *distinctive treatment* (note that this could also be described as *positive* distinctive treatment, yet more simply referred to here as distinctive treatment; see General Discussion for more on the idea of experiencing distinctly *negative* treatment). Distinctive treatment represents a collection of behaviors and other (non)verbal expressions coming from group members that signal to an individual that they possess, or have the potential to develop, particular qualities that are important to the group. Such treatment includes instances when other group members call upon an individual to provide ideas or some form of guidance that helps the group or its members, particularly when it requires the individual to employ a particular skill or base of knowledge. For example, in work organizations, distinctive treatment is reflected in instances when employees call upon another for guidance on how to troubleshoot a certain type of problem. Other examples include when a member of a religious group seeks advice from another member on how to resolve a moral dilemma, or when a nurse asks another nurse for guidance on handling a difficult situation (e.g., reaching out to a fellow nurse who is particularly adept at ‘difficult [intravenous] sticks,’ or another nurse who is adept at troubleshooting complicated sets of presenting symptoms, or another who is known for quickly developing rapport with flustered patients). We posit that when others call upon an individual to provide this type of group-relevant guidance, it conveys a message to the individual that they possess qualities that are valued by the group, ultimately implying that they hold a distinct level of admiration or standing in the eyes of other group members. These messages in turn guide the individual’s own (reflected, internalized) appraisal of their intragroup standing.

Conceptually, distinctive treatment focuses on the actions of other group members toward the individual, and not the individual’s own actions. This focus aligns with theory that suggests individuals’ appraisals of the self, including their perceived intragroup standing, is fundamentally determined by the actions of others toward them, rather than their own actions [4, 6] (or more fundamentally, [19, 20]). Thus, distinctive treatment includes instances where an individual is called upon by others to provide some form of guidance, but does not reflect how often the individual provides it (whether solicited or unsolicited). So while an individual’s own actions may play a role in shaping their perceived standing, we posit that the more potent determinant will be the actions of other group members toward the individual.

More broadly, expressions of distinctive treatment help to recognize ‘the individual within the group’–recognizing the individual for the particular skills, knowledge, experience or insights they possess, and that are seen as an asset to the group. This conceptualization aligns with theory on ‘role differentiation’ in groups [21] and the importance of having a degree of intragroup distinctiveness. Distinctive *treatment* adds to this line of theorizing by explicating a behavioral mechanism–a specific form of treatment–through which others in a group can communicate to a fellow member that they are recognized and appreciated for their particular qualities and the corresponding ‘roles’ that this enables them to play in the group. Notably, the magnitude of ‘distinctiveness’ that this type of treatment taps in to–and ultimately elicits within the individual (as an internalized, reflected appraisal of the self)–can be subtle. For example, instances at work where one is asked by colleagues for advice may not seem particularly distinguishing. Yet the act of seeking out one’s advice implies that the individual *has insights* that those other members may *not* have, highlighting a distinction between them. Of equal importance, the act of seeking out one’s advice highlights that those other members recognize and are eager to hear one’s insights, and thus informally place the individual in a differentiated role of ‘advisor’ (vs. ‘advice-seeker’). In this way, such treatment taps into situations whereby the target individual is subtly distinguished from those other members. Instances of distinctive treatment may also, or alternatively, distinguish the individual from other members not directly involved in the interaction yet whom also provide a meaningful basis for highlighting one’s distinguishing group-relevant qualities (e.g., being pulled aside by a supervisor and asked for advice may highlight that one is recognized for having certain insights that distinguish them from other colleagues, perhaps with the same formally designated role/position). In turn, experiencing this type of treatment is likely to elicit *within one* an internalized sense of distinct value and worth to the group–that is, a sense of intragroup standing, which similarly reflects a subtle yet healthy degree of *internalized* distinction (e.g., a sense of being looked up to within the group, which distinguishes one from those who are ‘looking up’).

It is important to note that providing a degree of distinction is not equivalent to providing a sense of *complete* distinction from every other member. In fact, several theories contend that complete distinction is undesirable; more modest differentiation is often more beneficial (for a review, see [22]). This means distinctive treatment is not limited to experiences of being sought out for advice or skills that no other member could provide (such instances reflect a particular and perhaps more rare instantiation of distinctive treatment, not a defining feature of it). Even if sought out for skills or advice that some others could provide, assuming not all others could provide it, the act of going to that individual will help them develop a sense of distinct value to the group (i.e., intragroup standing).

Finally, it is important to acknowledge that beyond certain forms of group-based treatment, individuals’ perceived intragroup standing can be shaped by other relevant sources of information. For example, in organizations, job titles and salaries can carry straightforward messages about individuals’ standing in the group. However, while these indicators are important (see control variables in several of the current studies) they are only found in certain groups (e.g., with formal structure). As such, they cannot explain individuals’ perceived standing in less formally structured groups (see Studies 5 and 6). Therefore, it is critical to consider whether other types of cues–embedded in the treatment coming from other group members during everyday interactions (i.e., expressions of distinctive treatment)–can be readily found in a range of groups, through which individuals’ perceived standing is shaped. Arguably it would be quite valuable to identify a set of cues that operate similarly across a range of groups, including large and small groups, those with well-defined structures (e.g., work organizations) and those that are more diffuse (e.g., racial/ethnic groups). The current research aims to explicate just that. It also provides an initial, readily adaptable measure of it (i.e., of distinctive treatment).

For further conceptual extensions and applications of distinctive treatment, see the General Discussion. This includes the role of distinctive treatment in promoting diversity and inclusion in groups, and how it relates to other lines of theorizing (e.g., optimal distinctiveness theory).

### Summary of predictions

**Fig 1:**
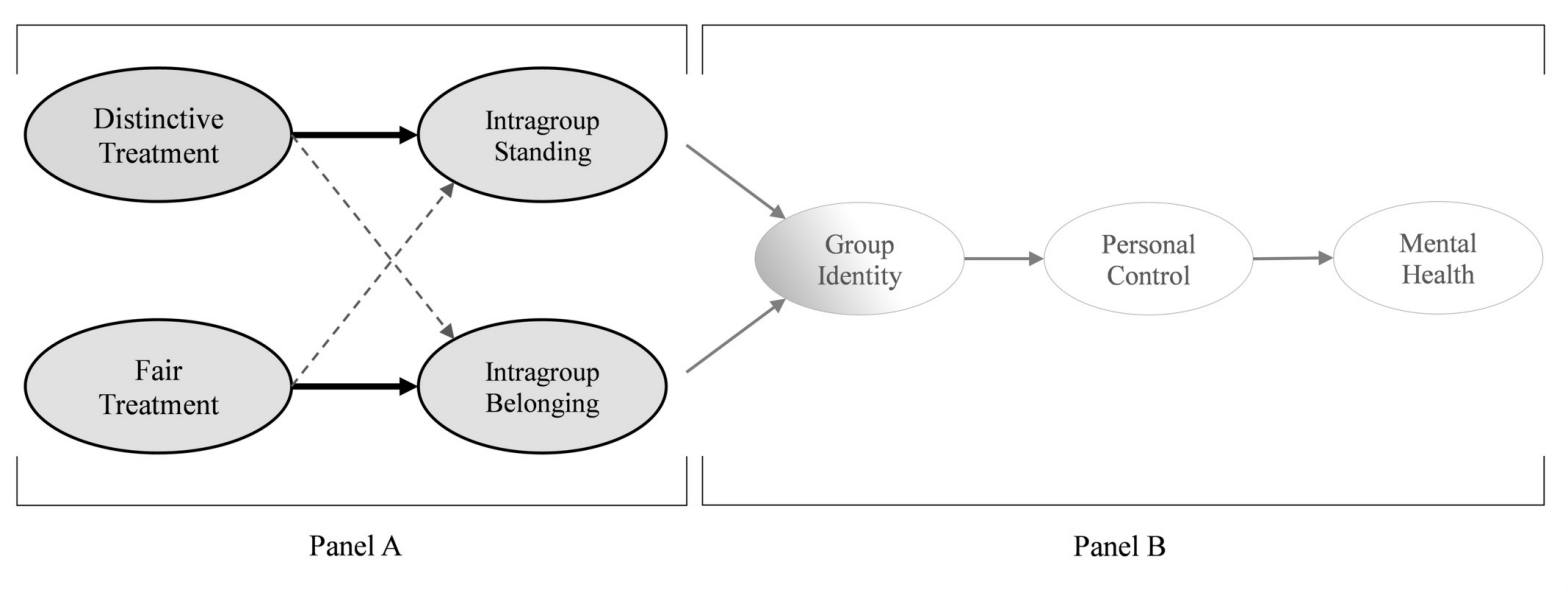
Hypothesized processes. Panel A outlines key hypothesized processes, explicating how two forms of group-based treatment–an oft-studied form, fair treatment, and a second form proposed here, distinctive treatment–differentially shape individuals’ perceived belonging and standing in groups respectively. These processes are tested in Studies 1–6. Panel B explicates a key implication of such treatment, for individuals’ mental health. This is explained through identity-based processes outlined in past work [27]. These more proximal processes are tested in Studies 4–6. Portions are shaded to illustrate how processes outlined in distinct literatures are brought together. The portion in grey reflects processes grounded in the procedural justice literature. The portion in white reflects processes evinced in the social cure literature.

Our hypothesized processes are depicted in Fig 1. We centrally posit that there are two forms of group-based treatment that differentially guide individuals’ understanding of their relationship to the group (Fig 1, Panel A). While fair treatment primarily communicates information about the extent to which individuals belong in the group, distinctive treatment primarily conveys information about individuals’ standing in the group.

At the same time, we propose some modest ‘cross-over’ effects (dashed lines, Fig 1). Regarding the fair treatment-standing link, this is because fair treatment is, in practice, not always equally distributed across group members. Therefore, if individuals are treated with dignity, it may to some extent signal that they are more highly regarded than some others in the group. Regarding the distinctive treatment-belonging link, this is because when others seek out an individual’s guidance they may not only consider whether the individual has relevant knowledge or skills, but is also likable and approachable. Therefore, when an individual is sought out for guidance they may receive a secondary message that they are generally liked and accepted by others, thus positively shaping their sense of belonging. By extension, and in line with previous theorizing [21], this suggests that distinctive treatment can provide individuals with a sense of intragroup standing while still enabling, if not secondarily promoting, their sense of belonging, because their distinctiveness focally supports group goals and benefits the collective. In this way, distinctive treatment is unlikely to interfere with the ability to develop a sense of belonging.

To further probe the hypothesized processes underlying distinctive treatment, we test whether experiencing it informs individuals’ sense of intragroup standing even after controlling for their own tendency to engage in group-serving behaviors. As our theorizing suggests, while individuals’ own actions should play a role in shaping their perceived standing [23], it is largely determined by other members’ actions toward them. Thus, experiences of distinctive treatment should explain individuals’ perceived standing even after accounting for their own group-serving behaviors.

#### Implications of fair and distinctive treatment for health

Our primary aim is to delineate a form of group-based treatment, distinctive treatment, and examine how it operates in conjunction with another oft-studied form, fair treatment (Fig 1, Panel A). Secondarily, we examine some of the downstream implications of these two forms of treatment, namely for individuals’ mental health (Fig 1, Panel B). To do so, we integrate insights from two lines of research–that on procedural justice and on social identity and health (the ‘social cure’ [24]). Work on procedural justice shows that when individuals feel a strong sense of belonging and standing in groups (as a function of how they are treated) it strengthens their psychological connection, or identification, with the group [5, 25, 26]. In turn, work on the ‘social cure’ shows that strong group identification enables individuals to feel that they can draw upon the support and strength of that group as they strive to overcome challenges and achieve important goals–an ability that fosters a sense of personal control over life, which ultimately promotes health [27]. Thus, when integrated with the current theorizing, these lines of research explicate how experiencing distinctive and fair treatment can translate into better mental health. This is illustrated altogether in Fig 1.

### Overview of current research

Across six studies, we examined these processes in three different types of groups. Following tests of our core hypothesized processes longitudinally and experimentally (Fig 1, Panel A; Studies 1–3), we tested some of the broader implications of distinctive treatment, specifically for individuals’ mental health (Studies 4–6; Fig 1, Panel B; all data collected between 2014 and 2019).

In Study 1a, we examined individuals’ experiences with distinctive and fair treatment in the workplace, testing whether each plays a unique role in predicting, respectively, their sense of standing and belonging in the organization (see Fig 1, Panel A). We also tested whether distinctive treatment predicts individuals’ perceived standing over and above a host of other indicators of intragroup standing.

Study 1b tested these processes longitudinally. Studies 2 and 3 established causality–manipulating distinctive treatment experiences in the workplace and testing its effect on individuals’ intragroup standing (and belonging). Study 3 also manipulated fair treatment to assess whether its primary effect is, in contrast with distinctive treatment, on individuals’ sense of belonging.

Studies 4–6 tested the broader set of hypothesized processes (Fig 1, Panel A+B). This enabled us to: (i) further test our key predictions regarding the independent roles of distinctive and fair treatment, (ii) illustrate the downstream implications of such treatment for individuals’ mental health, and (iii) demonstrate the applicability and importance of studying distinctive treatment in a variety of real-world groups. Building on Studies 1–3, Study 4 examined these processes is the workplace. Studies 5 and 6 tested these processes in more diffuse and less formally structured groups, including an undergraduate student community (Study 5) and in racial/ethnic minority groups (Study 6).

Studies 1–4 were grounded in a context where concerns about intragroup standing are a particularly important facet of group life (e.g., where there are often salient status structures). In part, this offered a conservative test of the role of distinctive treatment in predicting individuals’ perceived standing, because we could simultaneously account for other, more formal indicators of standing. We could assess whether individuals’ experiences with distinctive treatment (e.g., being sought out for advice) was truly an independent determinant of their perceived standing, or merely a byproduct of other indicators (e.g., being a supervisor). Studies 5–6 were situated in contexts where there are fewer structural indicators of standing and where such dynamics may be less salient (e.g., racial/ethnic groups). This provided opportunities to test whether distinctive treatment remains relevant and meaningful, even in such groups.

Overall, with these studies we aimed to extend previous work by explicating how individuals can come to feel that they not only “fit in” (intragroup belonging) but also “stand out” (intragroup standing) within a variety of real-world groups, which hinges on experiencing two distinct forms of group-based treatment. Additionally, we aimed to provide initial evidence that these two forms of treatment can translate into better mental health (e.g., less anxiety, depression). Across studies, we found highly consistent evidence supporting our hypotheses.

## Study 1a: Experiencing distinctive treatment in work organizations

Study 1a tested whether distinctive and fair treatment in the workplace play differentiated roles in predicting individuals’ perceived standing and belonging. We also tested whether experiencing distinctive treatment explained individuals’ intragroup standing over and above several other potential indicators.

### Methods and materials

Participants were 302 individuals employed at organizations across the US and UK, recruited via Prolific to complete an online study (*M*_*age*_ = 35.80, *SD* = 10.10, 48.3% female, 85.4% non-Hispanic white, 87.7% employed full-time, 44.7% held managerial/supervisory positions). Power analyses indicated the study was well powered. For details, see *S1 File*. This study was approved by the University of Exeter, College of Life and Environmental Sciences (CLES) Psychology Ethics Committee (approval for eCLESPsy000646; participant consent obtained electronically).

#### Distinctive treatment

Drawing on past insights [5, 7, 21], we developed four items to measure how often participants experienced distinctive treatment. Consistent with its theoretical foundations, items assessed the frequency of other employees’ behavior toward the participant, not the participants’ own behavior. Items began, “Thinking about the other employees you interact with in this organization (face-to-face, via phone, email, etc.), how often do they …?,” e.g., “ask you for help because of certain knowledge, skills or perspectives you have,” “look to you for guidance when they have a question or problem” (see Table 1 for full list). Items assessed how often they experienced this type of treatment coming from other employees, from 1 (*never*) to 5 (*very often*, α = .89). Items were averaged to form a composite, with higher values representing more frequent experiences of distinctive treatment.

**Table 1:** Study 1a, 4, 5 and 6 factor analyses demonstrating distinctions between distinctive and fair treatment, and between distinctive treatment and intragroup standing, across three types of groups (work organizations, student communities, racial/ethnic minority groups).

Items ^a^		Study 1a		Study 4		Study 5		Study 6	
Thinking about the other employees you interact with in this organization (face-to-face, via phone, email, etc.), how often do they …? *[never-very often]*									
*DT*	…ask you for advice	**.88**	-.07	**.85**	-.02	**.91**	-.06	**.93**	-.05
*DT*	…look to you for guidance when they have a question or problem	**.86**	-.01	**.88**	-.05	**.86**	-.02	**.87**	-.03
*DT*	…ask you for help because of certain knowledge, skills or perspectives you have	**.84**	-.03	**.85**	-.03	**.81**	-.01	**.69**	.04
*DT*	…ask you to share your opinions and ideas about things	**.65**	.19	**.68**	.16	**.52**	.23	**.82**	.04
*FT*	…treat you fairly	.05	**.81**	-.03	**.78**	-.00	**.75**	-.04	**.78**
*FT*	…show care for your well-being	.01	**.80**	.02	**.83**	-.06	**.93**	.00	**.78**
*FT*	…treat you with openness and Honesty	-.03	**.75**	.04	**.77**	.04	**.78**	-.04	**.72**
*FT*	…take your needs into Consideration	-.06	**.86**	-.02	**.84**	.03	**.88**	.10	**.53**
Items ^a^		Study 1a		Study 4		Study 5		Study 6	
Within this organization (among employees), I feel that I am… *[strongly disagree-strongly agree]*									
*STAN*	…looked up to	.05	**.87**	-.04	**.95**	.03	**.91**	.03	**.91**
*STAN*	…seen as a role model for others in the organization	.04	**.86**	-.02	**.88**	.02	**.91**	-.05	**.94**
*STAN*	…held in high regard	.15	**.73**	.02	**.83**	-.03	**.92**	.01	**.88**
*STAN*	…seen as a leader within this organization	n/a	n/a	.04	**.79**	.00	**.80**	.03	**.83**
*STAN*	…admired	-.12	**.97**	.01	**.87**	-.02	**.92**	n/a	n/a
*DT*	…ask you for advice	**.87**	-.04	**.89**	-.06	**.90**	-.05	**.93**	-.03
*DT*	…look to you for guidance when they have a question or problem	**.83**	.03	**.84**	.03	**.85**	.00	**.86**	.00
*DT*	…ask you for help because of certain knowledge, skills or perspectives you have	**.87**	-.05	**.83**	.01	**.77**	.07	**.62**	.15
*DT*	…ask you to share your opinions and ideas about things	**.63**	.16	**.73**	.04	**.64**	.00	**.86**	-.05

The table shows lambdas from rotated factor solutions derived from EFAs conducted using principal axis factor method and oblique rotation; While CFAs may be justified, EFAs provide a rigorous empirical test of factor independence, partly by allowing items to freely cross-load and not forcing a certain number of retained factors; n/a = not applicable (item not measured). DT = Distinctive Treatment; FT = Fair Treatment; STAN = Intragroup Standing.

^a^ Item wording from Study 1a. For a list of all study items, including adaptations for other group contexts (e.g., racial/ethnic minority groups), see *S1 File*.

Items were designed for use across various groups with minimal adaptation (see Studies 5–6). Conceptually, they aim to capture individuals’ experiences of being recognized for their group-relevant qualities–certain skills, insights and perspectives they bring to the group–thereby highlighting subtle differentiations between themselves and other members, which in turn elicits *within them* a modest, internalized sense of distinct value to the group (i.e., a sense of intragroup standing; see Introduction for a more detailed explanation). Empirically, as shown over multiple studies, the ability of these items to tap into experiences that highlight subtle differentiations between group members and ultimately elicit within one a degree of distinction is demonstrated by the fact that they play a prominent role in explaining individuals’ differing levels of standing within groups–even when tested alongside several other factors that also, independently, serve to differentiate group members (e.g., level of seniority, relative salary, number of employees under one’s supervision; see Table 2 and S4 Table in S1 File).

**Table 2:** Study 1a (work organization) regression analysis, distinctive treatment predicting intragroup standing over other relevant constructs.

	Model 1	Model 2
Group-serving behavior	.34***	.22***
Number of employees under one’s supervision	.13**	.12**
Salary (relative to others in org.)	.24***	.18***
Procedural fairness	.14**	.15**
Fair Treatment	.20***	.13**
**Distinctive Treatment**	---	.30***

Total *R*^*2*^ = .55, Δ*R*^*2*^ = .06, *F*(1, 291) = 37.70, *p* < .001.

Local effect size, Cohen’s *f* ^2^ = .13 (small-medium effect).

Standardized coefficients displayed;

** *p* ≤ .01;

*** *p* ≤ .001.

Study 1a *N* = 302 (employees in companies/organizations).

Full statistics for this regression analysis are in *S1 File*.

Regarding multicollinearity: All values for VIF ≤ 1.61, Tolerance ≥ 0.62.

Results virtually identical to analyses run with additional covariates (all non-significant; Δ*R*^*2*^ = .06, *F*(1, 265) = 36.73, *p* < .001): level of seniority (self-report), having a managerial/supervisory position, salary (raw/non-relative), years employed in: org.; current position; field/profession overall; age, gender, level of education, employment status (full- vs. part-time).

#### Fair treatment

We measured how often participants were treated fairly by other employees with four items (adapted [3, 15]). Items began, “Thinking about the other employees you interact with in this organization (face-to-face, via phone, email, etc.), how often do they …?,” e.g., “treat you fairly” (see Table 1 for full list). Items were rated 1 (*never*) to 5 (*very often*, α = .88). Items were averaged to form a composite, with higher values representing more frequent experiences of fair treatment.

#### Intragroup standing

We measured participants’ perceived standing in the organization with four items [2, 28, 29]. Items began, “Within this organization (among employees), I feel that I am…,” e.g., “looked up to,” “admired” (see Table 1 for full list). Items were rated from 1 (*strongly disagree*) to 7 (*strongly agree*, α = .93). Items were averaged to form a composite, with higher values representing greater perceived standing.

Unlike measures of group-based treatment, which gauge other employees’ behavior toward the participant (metric: frequency), intragroup standing assesses participants’ own internalized appraisals of the self (metric: level of self-applicability). Table 1 illustrates that this conceptual distinction is also borne out empirically, as evinced across multiple studies. These intragroup standing items also help tap into one’s internal, perceptible degree of intragroup distinction. For instance, to feel admired distinguishes one from those who are ‘admiring,’ to feel looked up to distinguishes one from those who are ‘looking up,’ and to feel like a role model or leader distinguishes one from those who are ‘following.’ Moreover, in line with theory on the benefits of having modest distinction (rather than complete or extreme distinction; for a review, see [22]), these items aim to assess relatively modest grades of distinction.

#### Intragroup belonging

We measured participants’ sense of belonging in the organization with three items [2]. Items began, “Within this organization (among employees), I feel that I am…,” e.g., “accepted for who I am” (see *S1 File* for full list). Items were rated 1 (*strongly disagree*) to 7 (*strongly agree*, α = .89). Items were averaged to form a composite, with higher values representing greater perceived belonging.

#### Control variables

To conservatively test the predictive strength of distinctive treatment, we measured several additional constructs that may predict individuals’ perceived standing in their organization, including more formal indicators (e.g., number of employees under one’s supervision). For a complete list, see Table 2. To also test the importance of individuals’ own behavior, we assessed how often participants engaged in group-serving behaviors [30] (e.g., “how often do you…do things over and above what is expected of you to help improve the organization?” 1 *never*– 5 *very often*; α = .85). For thoroughness, we also assessed individuals’ experiences with procedural fairness [31] (e.g., “Overall, regarding the procedures/policies your organization uses, to what extent… are these procedures applied consistently?;” 1 *to a small extent*– 5 *to a large extent*, α = .88). Compared to our measure of fair treatment, which assessed more interactional aspects of fairness within the group (i.e., other members are fair toward one), procedural fairness assessed more procedural aspects (i.e., policies used within the organization are fair toward one). For a detailed description of all control variables, see *S1 File*.

### Results

#### Distinguishing distinctive and fair treatment

We tested whether distinctive and fair treatment were empirically independent constructs by running an exploratory factor analysis using principal axis factor method and oblique rotation. Eigenvalues and the scree plot indicated a clear two-factor solution. All items loaded onto their appropriate factors without substantial cross-loadings. This indicated that expressions of dignity coming from group members (fair treatment) were discernably unique from expressions of interest and appreciation for an individual’s group-relevant qualities (distinctive treatment). Table 1 illustrates this distinction–including replication across Studies 4, 5 and 6, encompassing a variety of real-world groups.

#### Distinguishing distinctive treatment and intragroup standing

Using the same protocols, we tested and found that distinctive treatment and intragroup standing were independent constructs. Thus, distinctive treatment captures the frequency of other members’ behaviors toward one, while intragroup standing captures a key facet of one’s own internal, group-based appraisal of the self. See Table 1 for details.

#### Testing the robustness of distinctive treatment over other indicators of one’s standing

We assessed whether distinctive treatment predicted individuals’ perceived standing over other relevant indicators. We ran a hierarchical regression analysis with several indicators in the first step, including more formal indicators (e.g., number of employees under one’s supervision), one’s own engagement in group-serving behavior, and experiences of fair treatment. After controlling for these, we added distinctive treatment (Table 2).

Overall, these other indicators collectively and individually predicted individuals’ sense of workplace standing. Yet when distinctive treatment was added in the second step, it was not only significant but one of the strongest predictors (Total *R*^*2*^ = .55, Δ*R*^*2*^ = .06, *F*(1, 291) = 37.70, *p* < .001; local effect size, Cohen’s *f* ^2^ = .13). It is also informative that after adding distinctive treatment, the other indicators remained significant. This indicated that distinctive treatment was not simply a byproduct of these other indicators; there was something uniquely predictive in the experience of having others seek out one’s ideas and advice at work–beyond what could be explained by one’s supervisory position, salary, or even one’s own tendency to engage in group-serving behaviors.

#### Primary analyses

**Fig 2:**
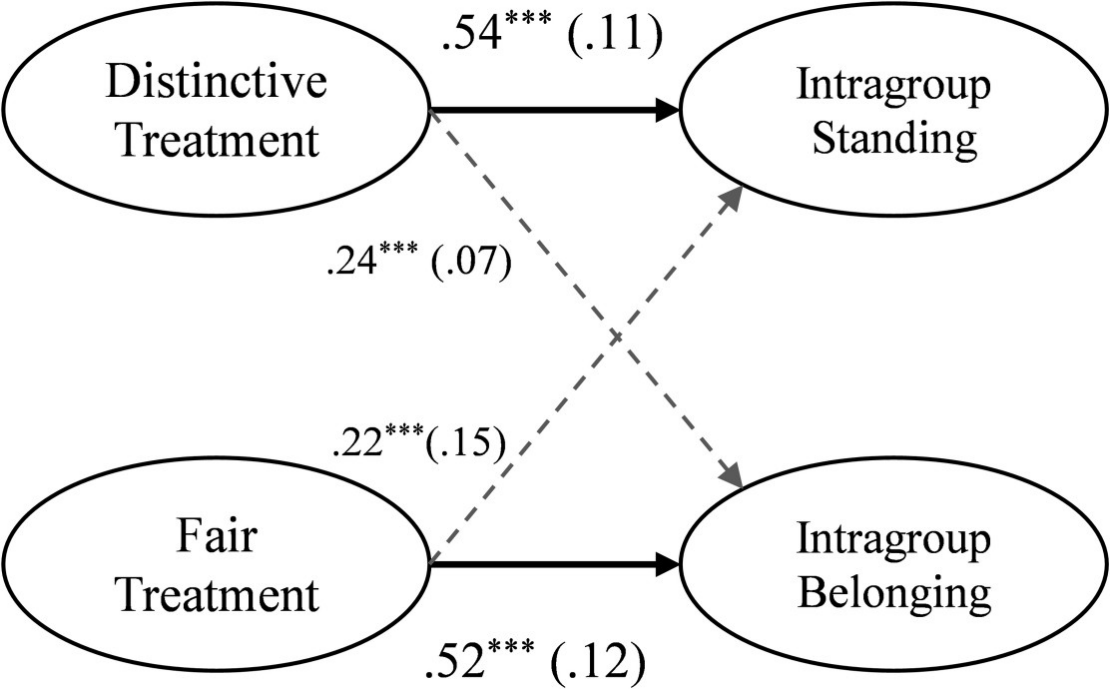
Study 1a results. Testing the roles of distinctive and fair treatment in predicting intragroup standing and belonging respectively, using SEM with standardized coefficients (standard errors). Factor loadings are omitted here, but all latent factors predicted their manifest indicators at *p* < .001. Correlations between distinctive and fair treatment (*r* = .41, *p* < .001) and intragroup standing and belonging (*r* = .28, *p* = .003) were specified. *** *p* ≤ .001.

We tested our key hypothesis–that distinctive and fair treatment play unique and differentiated roles in predicting individuals’ sense of standing and belonging in groups, respectively–using structural equation modeling (SEM) in EQS with robust maximum likelihood estimation [32, 33]. Latent factors were constructed to estimate each construct using their respective items as manifest indicators. See Fig 2 caption for more details (e.g., the correlation between fair and distinctive treatment).

Overall, the hypothesized model fit well, SB χ^2^ (84) = 143.0, *p* < .001, CFI = .97, RMSEA = .05 [.034, .062] and path coefficients supported each prediction (Fig 2). Individuals’ perceived standing was strongly predicted by their experiences with distinctive treatment, while fair treatment was a relatively weak predictor. By comparison, individuals’ sense of belonging was strongly predicted by their experiences with fair treatment, while distinctive treatment was a relatively weak predictor. Thus, distinctive and fair treatment played unique roles in explaining individuals’ perceived standing and belonging in their organization.

To further assess whether distinctive and fair treatment played unique roles, we compared the magnitude of their effects (*r*) via latent factor correlations, simultaneously accounting for the distinctive-fair treatment link [34]. As expected, distinctive treatment was a stronger predictor of intragroup standing than fair treatment, *z* = 3.84, *p* < .001. By comparison, fair treatment was a stronger predictor of belonging than distinctive treatment, *z* = 3.32, *p* < .001. This further illustrated the unique roles of distinctive and fair treatment in explaining individuals’ intragroup standing versus belonging. Note that Studies 4–6 replicated these findings across three different types of groups (see *S1 File* for detailed results).

#### Testing an alternative model

To further assess the importance of distinctive treatment, separate from fair treatment, we specified an alternative, fair treatment-only model in which distinctive treatment was absent. Results showed this model fit reasonably well and fair treatment predicted perceived standing and belonging. However, it accounted for very little variance in intragroup standing, *R*^2^_intragroup standing_ = .20. By comparison, the hypothesized model accounted for over twice that, *R*^2^_intragroup standing_ = .44, Δ*R*^2^ = .24, Cohen’s *f* ^2^ = .44 (large effect). Thus, accounting for individuals’ experiences with distinctive treatment provided a much stronger basis for understanding their perceived standing than fair treatment alone.

This alternative model was also tested longitudinally (Study 1b) and in other real-world groups (Studies 4–6). In each case, results mirrored those described here (see *S1 File* for detailed results). Thus, over time and in other types of groups, results consistently demonstrated that individuals’ experiences with distinctive treatment captured an indispensable piece of their intragroup interactions.

## Study 1b: Longitudinal test of distinctive treatment

Study 1a provided initial evidence supporting a model of intragroup relations that includes both distinctive and fair treatment as vital sources of information from the group. Study 1b extended this by testing the roles of distinctive and fair treatment longitudinally.

### Methods and materials

Study 1b included 182 participants from Study 1a, recruited for a follow-up survey approximately one year later (see *S1 File* for details). The Time 2 survey was an abbreviated version of the original, with the same measures of distinctive and fair treatment, intragroup standing, and belonging (all α ≥ .83). This study was approved by the University of Exeter, CLES Psychology Ethics Committee (approval for eCLESPsy000646; participant consent obtained electronically).

### Results

Using multilevel structural equation modeling in EQS, we tested whether distinctive and fair treatment predicted changes in intragroup standing and belonging over time. Latent factors were specified using each construct’s manifest indicators (all lambdas significant, *p* < .001). ICCs were ≥ .45, highlighting the importance of using a multilevel framework.

The hypothesized model fit the data well, both overall (RLS χ^2^ (168) = 157.3, *p* = .71, CFI = .97, RMSEA = .06 [.046, .072]) and in the within- and between-participants models separately (within-/between- models: average absolute standardized covariance residual = .03/.02; largest standardized residual = .11/.11). Moreover, path coefficients supported each prediction. Within individuals, those who experienced more distinctive treatment over time showed an increase in intragroup standing (*β* = .26, *p* < .001); to a lesser extent this was predicted by experiencing more fair treatment (*β* = .14, *p* = .003). Similarly, those who experienced more fair treatment over time showed an increase in belonging (*β* = .35, *p* < .001); to a lesser extent this was predicted by experiencing more distinctive treatment (*β* = .33, *p* < .001). Results of the between-participants model further supported predictions: distinctive treatment → intragroup standing [belonging], *β* = .62, *p* < .001 [*β* = .24, *p* = .01]; fair treatment → intragroup belonging [standing], *β* = .59, *p* = .003 [*β* = .25, *p* = .003]. Thus, distinctive and fair treatment played differentiated roles in explaining change in individuals’ intragroup standing versus belonging over time.

As in Study 1a, to further assess whether distinctive and fair treatment played unique roles we compared the magnitude of their effects. Results showed that across individuals and time, distinctive treatment was a stronger predictor of intragroup standing than fair treatment, *z* = 5.61, *p* < .001, and fair treatment was a stronger predictor of belonging than distinctive treatment, *z* = 4.82, *p* < .001. A similar pattern emerged in the within-participants model (intragroup standing: distinctive treatment was stronger than fair treatment, *z* = 1.42, *p* = .16; intragroup belonging: fair treatment was generally stronger than distinctive treatment, *z* = 0.29, *p* = .77), though the difference in magnitude of effects was smaller, particularly on intragroup belonging. Taken together, this indicated that distinctive and fair treatment played differentiated roles in explaining individuals’ intragroup standing versus belonging.

#### Testing an alternative model

To further assess the importance of distinctive treatment as a critical form of group-based treatment, separate from fair treatment, we specified an alternative model in which distinctive treatment was absent. As in Study 1a, results showed that this fair treatment-only model predicted perceived belonging (within-/between-models: *β* = .43, *p* = .003 / *β* = .69, *p* = .001) and standing (within-/between-models: *β* = .20, *p* = .002 / *β* = .47, *p* < .001). However, it explained relatively little variance on standing: within-/between-models: *R*^2^_intragroup standing_ = .04/.23. By comparison, the hypothesized model accounted for over twice that: *R*^2^_intragroup standing_ = .10/.56, Δ*R*^*2*^ = .06/.33, Cohen’s *f* ^2^ = .07/.75. Thus, accounting for individuals’ experiences with distinctive treatment was vital for understanding how their perceived standing at work changed over time.

## Study 1 discussion

Study 1 demonstrated that individuals’ experiences with distinctive and fair treatment in the workplace are both conceptually and empirically distinct (Table 1). Moreover, they play unique roles in explaining individuals’ sense of standing versus belonging in the group (e.g., Fig 2). When called upon by colleagues to provide guidance or utilize certain group-relevant skills (distinctive treatment), they are able to develop and maintain a stronger sense of standing within their workplace. Yet these experiences do far less to explain individuals’ feelings of belonging in the organization. Instead, it is when individuals are treated with dignity (fair treatment) that they feel a stronger sense of belonging.

Study 1 also demonstrated that experiences of distinctive treatment explain individuals’ perceived standing over and above a variety of other relevant indicators. Even when accounting for individuals’ own group-serving behaviors, other more objective indicators of standing (e.g., number of employees under one’s supervision), and their experiences with fair treatment, distinctive treatment was a strong predictor of intragroup standing. This suggests something powerful about the experience of seeing one’s value and worth through the eyes of other group members. It informs individuals’ perceived standing in a way that cannot be understood simply by considering their position, salary, or even their own efforts to go above and beyond to help the organization.

## Study 2: Experimental test of distinctive treatment

Study 1 supported predictions on the functioning of distinctive treatment, using data that captured individuals’ real-world workplace experiences, and, in the case of Study 1b, over time. To demonstrate causal effects, Studies 2 and 3 manipulated individuals’ experiences of distinctive treatment and tested its effects on their perceived standing and belonging. Study 3 built on this by manipulating distinctive *or* fair treatment, thereby enabling direct comparisons of their relative effects on intragroup standing and belonging. Studies 2 and 3 extended Study 1 by examining these processes in the workplace context.

### Methods and materials

Participants were 453 individuals employed at organizations in the US and UK, recruited via Prolific (*M*_*age*_ = 37.72, *SD* = 10.39, 53.4% female, 93.6% white/non-Hispanic, 75.7% employed full-time, 58.3% held managerial/supervisory positions). Power analyses indicated the study was well powered. For details, see *S1 File*. This study was approved by the University of Exeter, CLES Psychology Ethics Committee (approval for eCLESPsy000552; participant consent obtained electronically).

Participants were randomly assigned to one of two experimental conditions: distinctive treatment or control. After providing demographic information (e.g., age, employment status), they completed an autobiographical recall task, which served as the manipulation. This allowed us to maintain methodological rigor by implementing a truly randomized and double-blind manipulation, while also maintaining high ecological validity by utilizing individuals’ actual lived experiences–testing whether individuals’ real-world experiences with distinctive treatment can readily transform their perceived standing in the group. In the distinctive treatment condition, they described two past instances where a fellow employee or group of employees approached them for ideas, advice, or guidance on a work-related issue or sought them out for a particular skill or base of knowledge they possess that could help address a work-related matter. Participants in the control condition described the routes they take to and from work.

Participants then completed measures of intragroup standing (α = .93) and belonging (α = .85; see *S1 File* for a list of items). They also completed a recall check of the manipulation, and were asked how difficult it was to complete the recall task. The latter was to gauge the level of ease around completing the manipulation (e.g., how difficult it was to think of two instances of distinctive treatment). Participants also completed measures of other potential indicators of workplace standing (e.g., level of seniority).

### Results

Preliminary analyses tested if the conditions differed on any general or demographic variables. Results indicated they did not, though unsurprisingly those in the control condition found it easier to complete the recall task, *t*(451) = -8.79, *p* < .001.

#### Primary analyses

**Fig 3:**
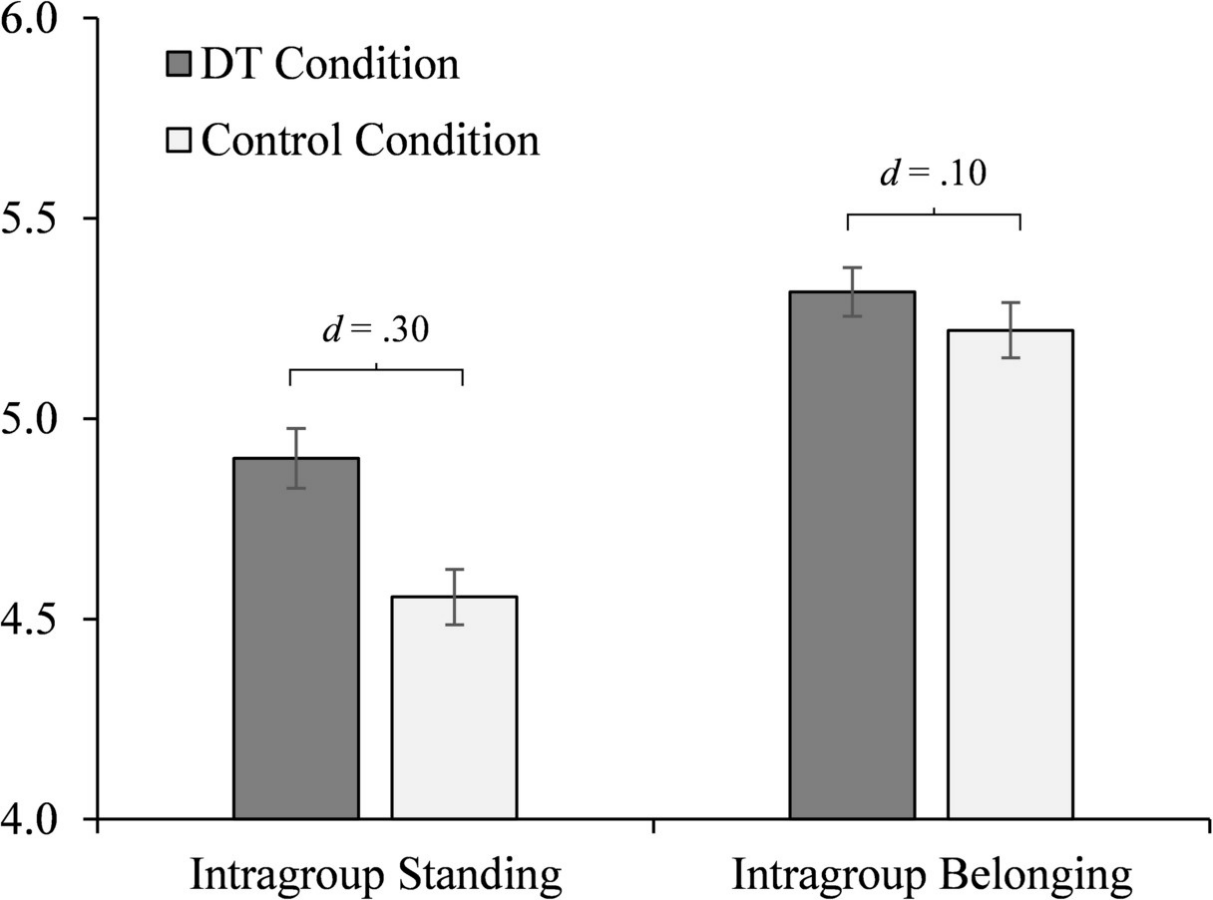
Study 2 results. Experimental effect of distinctive treatment (DT; compared to a control condition) on individuals’ sense of standing (*d* = .30) and belonging (*d* = .10, *ns*) in their work organization (measured on 1–7 scales; *N* = 453). Means represent estimates at the mean of the covariate. Error bars represent standard errors.

To account for the aforementioned difference in difficulty of recall task, we included it as a covariate in our primary analyses of (co)variance. As expected, these analyses of covariance showed that the distinctive treatment manipulation increased individuals’ perceived intragroup standing (*M* = 4.86, *SD* = 0.97; control condition, *M* = 4.58, *SD* = 1.20), *F*(1,450) = 9.32, *p* = .002, η_p_^2^ = .02 (adjusted values at mean of covariate: *M* = 4.90, *SD* = 1.15; control condition, *M* = 4.56, *SD* = 1.15, adjusted mean difference, *d* = .30; Fig 3; *d*’s calculated following [35]). In contrast, the manipulation did not affect individuals’ sense of belonging (*M* = 5.26, *SD* = 0.96; control condition, *M* = 5.27, *SD* = 0.97), *F*(1,450) = 0.96, *p* = .33, η_p_^2^ = .002 (adjusted values at mean of covariate: *M* = 5.32, *SD* = 1.00; control condition, *M* = 5.22, *SD* = 0.99, *d* = .10). Further analysis revealed that the effect of distinctive treatment on intragroup standing remained evident even when controlling for other relevant indicators (e.g., number of employees under one’s supervision, level of seniority), and when accounting for individuals’ sense of belonging–that is, controlling for any variance shared between individuals’ sense of belonging and standing, and/or any effect the manipulation had on belonging, thus more precisely isolating the effect of the distinctive treatment manipulation on intragroup standing, *F*(1,428) = 7.48, *p* = .01, η_p_^2^ = .02; adjusted mean difference, *d* = .22. Thus, overall, results consistently showed that distinctive treatment affected individuals’ intragroup standing.

## Study 3: Experimental manipulation of distinctive and fair treatment

Study 3 aimed to replicate and extend Study 2 by adding a third condition, manipulating fair treatment. The expanded design allowed us to not only test whether the effect of distinctive treatment was primarily on individuals’ sense of standing but also whether the effect of fair treatment was primarily on belonging. To provide a more precise and rigorous test of hypothesized effects, in primary analyses we examined the effect of distinctive treatment on intragroup standing while controlling for individuals’ sense of belonging (and any effect the manipulation had on it). By including it as a covariate we could isolate that aspect of individuals’ intragroup standing that was entirely distinct from (shares no variance with) their sense of belonging. If the effect of distinctive treatment remained significant after controlling for belonging, this would demonstrate that the effect is substantive, and not driven by the shared variance between standing and belonging. Similarly, we tested the effect of fair treatment on belonging, controlling for standing.

### Methods and materials

Participants were 427 employees at organizations in the US and UK, recruited via Prolific (*M*_*age*_ = 38.07, *SD* = 10.45, 63.9% female, 89.7% white/non-Hispanic, 77.8% employed full-time, 58.9% held managerial/supervisory positions). Power analyses indicated the study was well powered. For details, see *S1 File*. This study was approved by the University of Exeter, CLES Psychology Ethics Committee (approval for eCLESPsy000552; participant consent obtained electronically).

We randomly assigned participants to one of three experimental conditions: distinctive treatment, fair treatment, control. The procedure and measures mirrored Study 2, with the control and distinctive treatment conditions being the same. In the fair treatment condition, participants described two past instances in which a fellow employee or group of employees treated them in a way that was generally fair, honest, and considerate when dealing with a work-related issue, or showed consideration for them in the context of a work-related task. Participants then completed the same key measures as in Study 2: perceived intragroup standing (α = .93) and belonging (α = .84).

### Results

Planned group comparisons tested for differences between the control condition and each treatment condition. Preliminary analyses indicated participants across conditions were demographically alike, though as in Study 2 those in the treatment conditions (distinctive and fair) found it more difficult to complete the recall task compared to the control, *t*’s > 5.00, *p*’s < .001. To account for these differences, we included this as a covariate in subsequent analyses of covariance.

#### Initial test of effects

As in Study 2, the distinctive treatment manipulation affected individuals’ sense of intragroup standing (*M* = 4.88, *SD* = 1.17; control condition, *M* = 4.53, *SD* = 1.16), *F*(1,294) = 9.03, *p* = .003, η_p_^2^ = .03; adjusted mean difference, *d* = .36. It also affected their sense of belonging, though the effect was smaller (*M* = 5.55, *SD* = 0.86; control condition, *M* = 5.36, *SD* = 1.01), *F*(1,294) = 4.48, *p* = .04, η_p_^2^ = .02; adjusted mean difference, *d* = .25. By comparison, when examining the fair treatment and control conditions, the pattern of findings was reversed. The most prominent effect of fair treatment was on individuals’ sense of belonging (*M* = 5.67, *SD* = 0.85), *F*(1,289) = 14.61, *p* < .001, η_p_^2^ = .05; adjusted mean difference, *d* = .47. Its effect on intragroup standing was also significant but slightly smaller (*M* = 4.88, *SD* = 1.10), *F*(1,289) = 13.63, *p* < .001, η_p_^2^ = .05; adjusted mean difference, *d* = .46 (omnibus tests: intragroup standing, *F*(2,423) = 9.10, *p* < .001, η_p_^2^ = .04; intragroup belonging, *F*(2,423) = 7.44, *p* = .001, η_p_^2^ = .03).

#### Primary test of effects

Perceived intragroup standing and belonging are conceptually and empirically distinct but correlated. To better examine the unique effects of distinctive and fair treatment, we conducted analyses on each of the two dependent variables while controlling for the other. By including each as a covariate when examining treatment effects on the other, we isolated that aspect of each construct that is distinct from the other. Thus, these analyses more precisely isolated and therefore conservatively tested the hypothesized, differential effects of distinctive and fair treatment on intragroup standing and belonging respectively.

**Fig 4:**
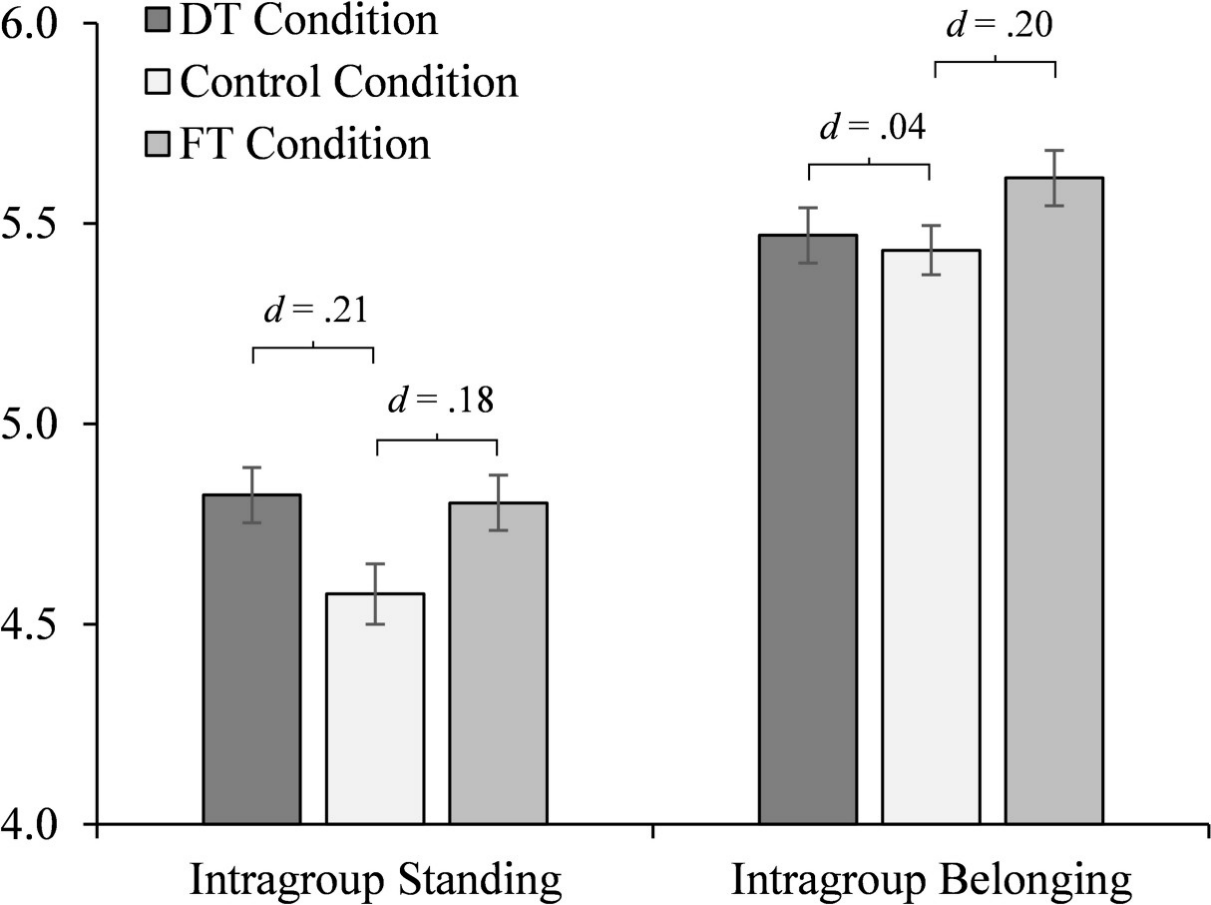
Study 3 results. Experimental effects of distinctive treatment (DT) or fair treatment (FT; each compared to a control condition) on individuals’ sense of standing (DT, *d* = .21; FT, *d* = .18, *ns*) and belonging (DT, *d* = .04, *ns*; FT, *d* = .20) in their work organization (measured on 1–7 scales; *N* = 427). Means represent estimates at the mean of covariates (as reported in main text). Error bars represent standard errors.

Results showed that the effect of distinctive treatment on intragroup standing held even when controlling for individuals’ sense of belonging (and any effect the manipulation had on it), *F*(1,293) = 4.63, *p* = .03, η_p_^2^ = .02 (adjusted values at mean of covariates: *M* = 4.82, *SD* = 0.96; control condition, *M* = 4.58, *SD* = 0.96; adjusted mean difference, *d* = .21; Fig 4). Yet the cross-over effect of distinctive treatment on belonging was no longer evident when intragroup standing was accounted for, *F*(1,293) = 0.16, *p* = .69, η_p_^2^ = .001 (adjusted values at mean of covariates: *M* = 5.47, *SD* = 0.78; control condition, *M* = 5.43, *SD* = 0.78, adjusted mean difference, *d* = .04). This pattern was reversed when examining the effects of fair treatment; its effect on belonging held when controlling for intragroup standing, *F*(1,288) = 4.09, *p* = .04, η_p_^2^ = .01 (adjusted value at mean of covariates: fair treatment condition, *M* = 5.61, *SD* = 0.82; adjusted mean difference [vs. control], *d* = .20). Yet the cross-over effect of fair treatment on intragroup standing was no longer evident when controlling for belonging, *F*(1,288) = 3.15, *p* = .08, η_p_^2^ = .01 (adjusted value at mean of covariates: fair treatment condition, *M* = 4.80, *SD* = 0.99; adjusted mean difference [vs. control], *d* = .18; omnibus tests: intragroup standing, *F*(2,422) = 3.47, *p* = .03, η_p_^2^ = .02; intragroup belonging, *F*(2,422) = 1.86, *p* = .16, η_p_^2^ = .01). Thus, distinctive treatment most prominently and robustly affected individuals’ sense of standing. Fair treatment most prominently affected their belonging.

## Studies 4–6: Testing distinctive treatment across different social groups, and its implications for health

Studies 4–6 built on Studies 1–3 in two key ways. First, they tested the downstream implications of distinctive and fair treatment for individuals’ mental health (anxiety, depression). Fig 1 outlines these processes (Panel B). Past work provides causal evidence for many of these downstream processes [3, 27], which Studies 4–6 built on by explicating their antecedents (Fig 1, Panel A)–antecedent processes that were themselves tested experimentally and longitudinally in Studies 1–3. Thus, Studies 4–6 extended past theory on social identity and health by examining its antecedents (fair and distinctive treatment) and, therein, illustrating the broader implications of such treatment for individuals’ mental health.

Second, Studies 4–6 tested whether distinctive treatment (and these other distal processes) function similarly across a multitude of real-world groups. Study 4 tested these processes in the workplace (a direct extension of Studies 1–3). Study 5 examined them in the context of a student community, and Study 6 in the context of racial/ethnic minority groups. Like the workplace, these other group contexts represent ones in which individuals often spend a lot of time. However, they differ in the degree of formal structure, size, and diffuseness. Testing the role of distinctive treatment in these contexts was therefore an opportunity to assess just how broadly applicable it is for understanding individuals’ perceived standing, identity, and health. Studies 5 and 6 also provided an opportunity to assess whether our measure of distinctive treatment was reliable across these different group contexts.

### Methods and materials

A detailed description of methods and materials are in *S1 File* (e.g., participants, measures, detailed results). In short, Study 4 participants were 494 individuals employed at organizations across the US (*M*_*age*_ = 34.66, *SD* = 10.40, 44.8% female, 78.8% white/non-Hispanic). Most worked full-time (88.3%) and nearly half held supervisory positions (45.6%). Study 5 participants were 190 undergraduates from a large US public university (*M*_age_ = 19.23, *SD* = 1.30, 72.9% female). Study 6 participants were 322 US-born Asian/Asian American and Latinx/Hispanic students from a large US public university (*M*_age_ = 20.70, *SD* = 2.13, 73.0% female; 56.2% Latinx/Hispanic). All three studies were approved by the University of California, Los Angeles Institutional Review Board (approval for 15–001793 [Studies 4–5], 13–001727 [Study 6]; participant consent obtained electronically).

### Results

For brevity, here we provide an overview of key findings from Studies 4–6.

#### Empirically distinguishing constructs

In all three studies (Studies 4–6), EFAs indicated that distinctive and fair treatment were independent constructs, as were distinctive treatment and intragroup standing (Table 1).

#### Testing the robustness of distinctive treatment over other indicators of one’s standing

Across all three studies/group contexts, hierarchical regression analyses showed that distinctive treatment predicted individuals’ intragroup standing over and above several other relevant indicators (S4-S6 Tables in S1 File).

#### Primary analyses

**Fig 5:**
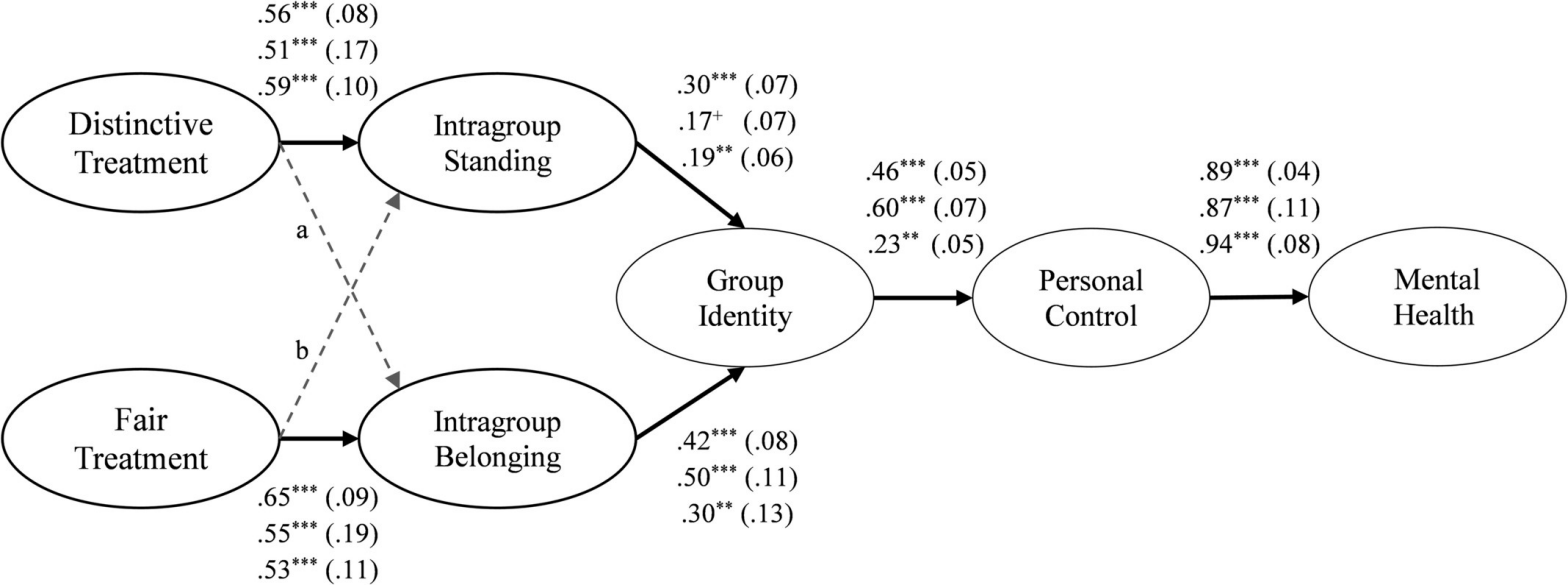
Results of Study 4 (work organizations), Study 5 (student community), and Study 6 (racial/ethnic minority groups). Results of each study demonstrated the differentiated roles of distinctive and fair treatment and the downstream implications of such treatment for individuals’ group identity and health. Standardized path coefficients (standard errors) are shown. Study 4/5/6 path coefficients are listed top/middle/bottom. Factor loadings are omitted here though all predicted their respective manifest indicators (*p* ≤ .001). Correlations between distinctive and fair treatment (Study 4/5/6, *r* = .47/.54/.44, *p* ≤ .001) and intragroup standing and belonging (Study 4/5/6, *r* = .39/.29/.30, *p* ≤ .001) were also specified. *** *p* ≤ .001; ** *p* ≤ .01; ^+^ *p* = .06; ^†^ *p* = .10. ^a^ direct path coefficient (Study 4, 5, 6): .19*** (.06), .18^†^ (.11), .29*** (.07); ^b^ direct path coefficient (Study 4, 5, 6): .26*** (.10), .16^+^ (.25), .16** (.15).

Across studies, results of SEM analyses consistently supported predictions (Fig 5). In each study, the model fit the data well; Study 4: SB χ^2^ (220) = 453.1, *p* < .001, CFI = .96, RMSEA = .05 [.042, .054]; Study 5: SB χ^2^ (220) = 289.3, *p* = .001, CFI = .97, RMSEA = .04 [.027, .053], Study 6: SB χ^2^ (199) = 341.2, *p* < .001, CFI = .95, RMSEA = .05 [.039, .056]. Results also showed that individuals’ perceived standing was most strongly predicted by distinctive treatment, while their sense of belonging was most strongly predicted by fair treatment. Individuals’ sense of standing and belonging subsequently predicted stronger group identification, and in turn a greater sense of control over life and better mental health.

Thus, results indicated that within a variety of real-world groups distinctive and fair treatment played differentiated roles in explaining individuals’ intragroup standing and belonging. Moreover, a strong sense of standing and belonging had downstream benefits for mental health (anxiety, depression).

## General discussion

This research extends previous theory in the procedural justice literature by explaining how individuals can come to feel that they not only “fit in” (intragroup belonging) but also “stand out” (intragroup standing) within a variety of important, real-world groups. Both are vital to developing a healthy sense of self, and, as shown, hinge on experiencing two distinct forms of group-based treatment.

Indeed, a core contribution of this work is to delineate these two separate forms of treatment. Alongside the oft-studied form, fair treatment, we outline another: *distinctive treatment*. As demonstrated, both inform individuals’ group-based appraisals of the self. Yet they convey different information, and thus shape distinct aspects of individuals’ sense of self. Whereas fair treatment focally communicates a sense of belonging in the group, distinctive treatment provides individuals a sense of distinct value and worth to the group (intragroup standing) by highlighting their particular group-relevant qualities. Thus, while past research has focused on the benefits of experiencing fair treatment in groups, our work demonstrates that experiencing distinctive treatment is also a critical facet of individuals’ intragroup relations. Moreover, we integrate recent theory on social identity processes [27] to explain how these two forms of group-based treatment can operate in tandem to promote greater mental health.

Across six studies, we evinced support for these processes. Results demonstrated that distinctive and fair treatment indeed play unique roles in shaping individuals’ sense of standing and belonging in groups, respectively. Moreover, in probing the importance of individuals’ experiences with distinctive treatment, results demonstrated that its influence on individuals’ standing persists even after taking into account a variety of other relevant factors. This included individuals’ own group-serving behaviors, their experiences with fair treatment, and a host of other indicators of standing (e.g., holding a supervisory position at work). Thus, overall, these studies demonstrate the importance and potency of experiencing distinctive treatment in shaping individuals’ sense of self.

We also demonstrate that these processes operate within a range of different groups–from those that are more structured (work organizations) to more diffuse groups (e.g., racial/ethnic minority groups). Finding that individuals in each context had meaningful experiences of distinctive treatment illustrates its broad utility, and thus the importance of studying distinctive treatment (also see [36, 37]). This also suggests the study of distinctive treatment, and our measure of it, may be useful to consider in other research areas, from organizational processes to minority mental health.

### Theoretical contributions and real-world applications

#### Delineating two forms of group-based treatment

Past research reliably demonstrates that when individuals are treated fairly in a group, it impacts how they view themselves in relation to that group [3, 5, 7, 8]. In the current research we examine what, specifically, these fair treatment experiences foster–a sense of belonging, a sense of standing, or both equally. Across six studies, results consistently demonstrated that experiencing fair treatment primarily fostered individuals’ sense of belonging in groups.

This highlights a critical point, both theoretically and practically: experiencing fair treatment is important, but not sufficient. It is limited in its ability to provide individuals with a sense of being admired and looked up to in the group (see, e.g., results of the alternative model in Studies 1a, 1b, 4–6). Fortunately, distinctive treatment provides exactly that. It highlights the qualities, skills, and insights that individuals possess, and that are valued by the group.

The current research provides conceptual and empirical distinctions between these two forms of treatment (see, e.g., Table 1). Yet it is informative that fair treatment had some bearing on individuals’ sense of standing (see dashed lines in Fig 1). This is important because it explains how amidst conceptual and operational ambiguity in past work on fair treatment, with some pointing to it as a source of belonging and other work showing it communicates standing, that work could evince a reliable effect–because to some extent it affects both, though as shown here, to different degrees. Perhaps more importantly, in the current research we show that even after controlling for fair treatment, distinctive treatment consistently produces a reliable, and more potent, effect on individuals’ sense of standing. Thus, by delineating these two forms of treatment we provide both theoretical clarity on past work and an advancement in our ability to explain how individuals discern two key facets of group life–a sense of belonging and intragroup standing.

Conceptually delineating these two forms of treatment may also deepen our understanding of research on civility, which reflects a quality of treatment with conceptual parallels to that of fair treatment (e.g., expressions of politeness, dignity; though not conceptually identical) [38, 39]. Yet civility has at times been operationalized to straddle notions of both fair and distinctive treatment (e.g., [37; Study 2]). With the distinction between these two forms of group-based treatment in mind, it may be possible to more precisely discern what specific enactments of ‘civility’ coming from fellow group members shape one’s sense of intragroup belonging versus standing–or similarly, in line with previous civility research, what specific enactments of civility displayed by a given member engender perceptions of them as warm versus competent in the eyes of fellow members.

It is worth noting that there is an oppressive history tied to the labelling of behavior as “civil” (versus not “civil”). We do not encourage the use of this term. It has been used to dehumanize racial minorities and delegitimize affiliated civil rights movements, in part by labelling collective actions and related behaviors undertaken to attain greater equality, and the individuals enacting them, as not “civil” [40, 41].

#### Reflected appraisals and intragroup standing

Past work on the antecedents of intragroup standing has tended to focus on the role of individuals’ own actions (e.g., making self-sacrifices to help the group [23]). In the current research we find that while individuals’ own actions play a role in shaping their perceived standing in the group, it is predominantly determined by other group members’ behaviors toward them. Thus, seeing their value and worth reflected through the actions of fellow group members plays a critically important and independent role. In fact, across several studies we accounted for individuals’ own group-serving behaviors (doing things beyond what was expected of them to help the group), alongside other relevant indicators of standing, and consistently demonstrated that distinctive treatment was still a reliable and particularly strong determinant of their perceived standing. This suggests something powerful about an individual seeing their value and worth to a group affirmed through the actions of other members–something that cannot be understood simply by considering an individual’s position in an organization, their salary, or even their own efforts to serve the group.

#### Groups as both a source of belonging and differentiation

Theory in the social identity tradition [42, 43] has focused on the importance of groups for fostering individuals’ sense of belonging and cohesion with the group. Much less attention has been paid to the idea that groups can also, complementarily, serve to differentiate people *within* the group. Indeed, some suggest that because a sense of belonging serves to deindividuate or depersonalize group members, intragroup individuation may be antithetical. However, more recent theorizing in this tradition suggests groups can in fact provide both a sense of belonging *and* individual distinctiveness [21]. One way this can occur is through ‘role differentiation,’ which allows individuals to feel distinct from others in the group while maintaining a sense of belonging and cohesion because their distinctiveness is rooted in serving shared group interests.

The current research helps explain how this type of group-serving distinctiveness actually occurs. That is, an individual’s sense of differentiation emerges when others in the group express distinctive treatment toward them–treating them in ways that acknowledge their particular qualities. Moreover, as our theorizing on distinctive treatment suggests, because such expressions validate those qualities that serve the group, they are not antithetical to individuals’ sense of belonging and connection to the group. If anything, as we show, such treatment serves (secondarily) to *enhance* individuals’ sense of belonging. Thus, the current research adds to more recent theoretical developments in the social identity tradition by explicating the ways in which groups can complementarily provide members with a sense of belonging balanced with a degree of differentiation within the group.

Notably, this perspective also has some parallels with the idea in optimal distinctiveness theory [44] that individuals seek a balance between feeling differentiated from others and a sense of inclusion/belonging. However, our perspective differs in its emphasis in suggesting that–and explaining how–individuals can derive both a sense of belonging and distinction from *intragroup* relations (also see [21]; i.e., a sense of distinction need not depend on *intergroup* relations/comparisons). Our focus also differs in its main aims: to explain how two forms of group-based treatment affect two different, theoretically-derived aspects of individuals’ group-based appraisals (perceived belonging and standing), in line with theory in the procedural justice literature [4–7]. While the current research may also speak to other lines of theory, including on basic human needs or identity-based motives–theories that range in their articulation of one core motive (e.g., status [45]), to two (similarity, distinctiveness [44]), to three (relatedness, autonomy, competence [46]), to six (belonging, distinctiveness, meaning, self-esteem, continuity, efficacy [47])–it is a direction for future research to rigorously assess (empirically, and thus provide meaningful and reliable insights, theoretically) as to how and to what extent expressions of fair and distinctive treatment within a given group may help satiate these various possible motives, or how these other various frameworks precisely map onto the notions of intragroup standing and belonging as conceptualized in the procedural justice literature.

#### Utilizing distinctive treatment to promote diversity & inclusion

While the current findings are important to consider for theoretical reasons, they also have important practical implications. This includes how organizations, educational institutions and other groups might utilize expressions of distinctive treatment to more effectively promote diversity and inclusion in groups. For instance, our theorizing on distinctive treatment, and our empirical evidence, indicate that experiencing distinctive treatment does not foster any sort of divisiveness but in fact secondarily *promotes* a sense of inclusion and belonging among group members, in part because the distinct value that this treatment affords individuals is rooted in those qualities that they can ultimately be used to serve and help promote the group’s shared goals and interests.

We further contend that while not everyone in a group may experience distinctive treatment, there are a wealth of opportunities to ‘distribute’ it. For example, with well-developed systems of mentorship built into a group (e.g., in a work organization, school or university), there can be an abundance of opportunities for members to become role models, or a go-to source of information and guidance for a collection of individuals within the group [48]. Moreover, to the extent that a group has a variety of qualities, skillsets, bases of knowledge and perspectives that are relevant and valuable to the group, different individuals can be led to feel distinctly valued with respect to these different qualities, skillsets, bases of knowledge, and perspectives–further enhancing the bandwidth for distributing distinctive treatment. For instance, even among a group of nurses on a unit who all have similar if not identical job titles, training and education, different individuals may be recognized and valued for different group-relevant qualities. One nurse may be the go-to person for help with starting difficult IVs, while another may be a key source of guidance for troubleshooting complicated sets of presenting symptoms. Still another nurse (or select few nurses) may be recognized for their ability to quickly develop rapport with patients, and still another for their ability to be an effective mentor and guide for new nurses (or nursing assistants) on the unit. Thus, we contend that expressions of distinctive treatment can serve to embolden a wealth of members’ perceived value and worth to the group, and simultaneously play a part in promoting their sense of inclusion and belonging.

Altogether, while additional research is needed, the current evidence and theorizing suggest that alongside fair treatment, distinctive treatment can be asset rather than a hindrance to promoting diverse and inclusive groups. In fact, distinctive treatment may be a vital yet overlooked element to diversity-promotion strategies, as it provides a path for recognizing and appreciating, and thus *encouraging*, a diversity of ideas, insights, knowledge and skills that individual members can bring to the group. Therein, it suggests how organizations, educational institutions and other types of groups could help members develop a sense that they not only “fit in” but also “stand out” in ways that are valued by and contribute to group’s collective goals.

### Limitations and future directions

We set out to establish and test the function of two key types of treatment that individuals experience in groups, and their effects on individuals’ sense of self. Studies 4–6 further evinced the robustness of these processes across different types of groups, while also testing a broader model that explicates their downstream implications for mental health. While this broader model has value–empirically because it is robust across different types of groups, theoretically because it integrates multiple perspectives including from the procedural justice and the social cure literatures–the current tests of these more distal processes utilize cross-sectional data, precluding causal claims. However, these downstream processes are experimentally tested and supported in previous work [8, 27, 49]. Still, it will be useful to further test the directionality of these pathways altogether in future research.

Future tests of this model should also consider other relevant processes that shape mental health, including those that may be more group-specific. For instance, while we find support for this model across several real-world groups, including racial/ethnic minority groups, there may be other key forces that shape minority group members’ sense of personal control and mental health in particular. This includes minority individuals’ experiences with racial/ethnic discrimination [50]. Consistent with this idea, Fig 4 shows that while the magnitude of certain parameters were similar across all three studies/group types (e.g., distinctive treatment → intragroup standing), there was greater variation in the size of other parameters. Most notably, Study 6 indicated that racial/ethnic minority group identification was a less potent predictor of personal control, compared to identification in the other studies/group contexts (work organizations, student communities; in Study 6, the parameter was at most half the size). This may be because racial/ethnic discrimination also plays a prominent, yet negative, role in shaping minority group members’ sense of personal control. In fact, research indicates that having stronger racial/ethnic identification increases one’s likelihood of recognizing and experiencing discrimination [28, 51, 52], which may in turn reduce their sense of personal control over life if not also their mental health [29, 53]. This suggests the more modest identification-personal control link we found for racial/ethnic minorities may be because it is simultaneously underpinned by a negative indirect effect of increased racial/ethnic discrimination.

Current tests of this model also prompt other intriguing questions about the nature of these processes within different types of groups. For instance, while we found that this model fit well across all three types of groups, results also indicated that in the context of an individual’s student community (Study 5), a sense of belonging was a particularly potent predictor of group identification (compared to intragroup standing). This suggests that in some groups individuals may place a premium on feeling included (belonging), more than looked up to (standing), at least when it comes to determining the strength of their group identification. Future studies might further examine if and when individuals’ perceived belonging versus standing may be markedly more or less important (e.g., for group identification), including as a function of the group type.

The current studies relied on self-reports of perceived standing in groups, and thus has the potential for single-source bias. Importantly however, individuals’ self-reports of intragroup standing are rather accurate insofar as they parallel other group members’ perceptions of their standing [54]. Nevertheless, going forward it will be valuable to assess intragroup standing using other approaches (e.g., peer evaluations, which may also be less influenced by a target individuals’ own trait-like features; e.g., levels of neuroticism). Our work similarly used self-reports of distinctive and fair treatment. Arguably however, individuals’ own perceptions of how they are treated matter most, because it reflects one’s true experience (even if subjective) and thus has the most potent effect on one’s self-concept (if not also on one’s health, just as one’s subjective position in society is a better predictor of health than their ‘objective’ position [55]). This also aligns with theorizing on reflected appraisals, which highlights the importance of individuals’ own internalization of their interactions and experiences with others for understanding the self. Still, it will be important to extend the current research by examining others’ expressions of distinctive and fair treatment toward the target (also see [36, 37]).

Future experimental work should also test whether some processes examined in the current studies have bidirectional effects, including between distinctive treatment and intragroup standing. While our hypothesized causal direction is theoretically derived and empirically supported (by our experimental studies; see also [37]), it is possible that having higher perceived standing might also feed back into experiencing more distinctive treatment. For instance, if higher perceived standing emboldens one’s sense of ambitions at work and motivates them to learn new group-relevant skills or knowledge, other members may in turn seek out that individual more often for pertinent advice or guidance.

#### Impact of distinctive treatment from different sources

Complementing the current research, future work on distinctive treatment should consider whether the source of distinctive treatment differentially impacts an individual’s sense of intragroup standing. For example, in an organizational context, expressions from authority figures may hold more weight in comparison to those from peers or subordinates, and thus do more to impact an individual’s sense of standing. There may be important compensatory effects to consider as well, when for example distinctive treatment is expressed by peers but not authority figures (or vice versa) [56].

#### Individuals’ experiences in more broadly or narrowly defined groups

Future research might also complement the current studies by examining the effects of experiencing distinctive treatment in more broadly or narrowly defined groups. For instance, we examined individuals’ experiences within their own undergraduate community (Study 5), employing multiple strategies to ensure participants focused on this particular group referent, and found that it is quite relevant for understanding their appraisals of the self and mental health. Yet it may also be worth considering undergraduates’ distinctive treatment experiences within more broadly defined groups (e.g., the broader university community including faculty, staff, graduate students) or more narrowly defined ones (e.g., undergraduates specifically within one’s own major).

#### Other manifestations of distinctive treatment

In the current research we focused on a manifestation of distinctive treatment whereby others in a group solicit one’s guidance, knowledge, ideas or skills. There are likely other ways to express distinctive treatment, including more explicit verbal expressions (e.g., other members going to a particular individual and stating, “we really value the particular skills you bring to this organization”) and more subtle, nonverbal expressions (e.g., facial expressions suggesting interest in hearing more about one’s ideas or perspectives; such as raised eyebrows and enthusiastic head nods). These potential means of expressing distinctive treatment will be important to assess in the future, to help flesh out the multiple ways it gets expressed in everyday interactions.

It will also be important to consider instances when individuals’ group-relevant contributions are ‘recognized’ per se, but in such a manner that it has no discernable benefit for their sense of intragroup standing. For instance, if an organization sends everyone in the group a generic “thank you for your hard work” email, this might seem like recognition of individuals’ contributions, but may not shape perceived standing. This is because its universal or group-wide distribution may communicate to individuals that it is mainly an expression of basic equality principles–organizational efforts to ensure that *everyone* is (equally) recognized–rather than recognition of a particular individual’s specific group-relevant qualities. Indeed, this is quite different from being recognized for one’s particular skills or insights through instances where other group members seek them out (e.g., via requests for advice), as in the current research. This latter form of treatment is not only more individualized but also more *active*–others soliciting and looking to utilize an individual’s insights. Going forward, it will be important to examine the degree to which the potency of distinctive treatment hinges on it being an active solicitation (vs. ‘non-active’ expression; e.g., going to a particular individual and simply stating appreciation for the insights they bring to the group) and/or individualized versus more generic, including instances where it is so generic or universally distributed that it conveys more basic principles of ‘equality recognition’ rather than ‘individual recognition’ and is thus arguably no longer an expression of distinctive treatment (as described in our conceptualization of distinctive treatment, this type of treatment focally helps ‘recognize the individual within the group’).

#### Experiencing distinctly negative treatment

Our focus has been on individuals’ experiences with *positive* distinctive treatment. Going forward, it will be important to consider the implications of being treated in a distinctively *negative* way. This might include instances when others in a group highlight an individuals’ particularly negative group-relevant features (e.g., calling attention to an individual’s marked lack of experience, perspective or ability around certain group-relevant matters). It might also include instances when others in a group *avoid* a particular individual when seeking guidance on an issue despite knowing the individual has insights to offer, or when others in a group readily disregard or criticize an individual’s ideas or perspectives as though they are not valuable or useful.

One possibility is that this type of treatment represents the conceptual flip-side of same distinctive treatment coin. It simply has negative effects on individuals’ sense of standing in the group. Another possibility is that this type of treatment is conceptually distinct. Compared to positive distinctive treatment, negative distinctive treatment might represent a more ambiguous type of treatment. For instance, when others in a group avoid a particular individual when seeking guidance on an issue, that individual might infer that they receive this type of negative treatment because they are not seen as having any unique or valuable qualities to offer–communicating low intragroup standing–but they might also infer that they *are* seen as having valuable qualities, but still receive this negative treatment because others simply do not *like* them and so choose not to go to them for guidance–communicating low intragroup belonging. Thus, there may be more attributional ambiguity surrounding certain experiences of distinctly negative treatment and so its implications for individuals’ sense of standing and belonging may differ.

## Conclusions

Despite a seemingly prominent view that what organizations, institutions, and other groups need to most critically offer their members is fairness (dignity, and unbiased treatment), the current research suggests this type of treatment is important, but not sufficient. For individuals to develop a strong and healthy sense of self, which includes a feeling of belonging but also a sense of distinct value and worth to the group, a group needs to consider how to recognize and convey appreciation for what its different members bring to the table. Therefore, it is critical to acknowledge that embedded within those everyday interactions among group members there is not only an opportunity to convey fairness, but also an opportunity to convey interest and appreciation for one’s particular group-relevant qualities, skills, and ideas–that is, an opportunity to provide distinctive treatment. These two forms of treatment work in tandem to help individuals develop a healthy sense of self.

## Supporting information

S1 File Detailed study information. Supporting information includes additional pertinent references.
(DOCX)
