# Supplementary material for: Being treated fairly in groups is important, but not sufficient: The role of distinctive treatment in groups, and its implications for mental health
Source: PLoS One. 2021 May 14;16(5):e0251871. doi: 10.1371/journal.pone.0251871 (PMC8121286; doi:10.1371/journal.pone.0251871)
Supplement: S1 File — Supporting information includes additional pertinent references. (DOCX) [file pone.0251871.s001.docx]

**Studies 1-3: Additional information**

**Study 1a participants**

Participants were 302 individuals employed at organizations across the US and UK (*M_age_* = 35.80, *SD* = 10.10, 48.3% female, 85.4% non-Hispanic white, 4.3% East Asian [e.g., Chinese, Japanese, Korean], 3.0% Black [e.g., Caribbean, African], 3.0% Multiracial, 2.3% Latinx/Hispanic, 1.0% South Asian [e.g., Indian, Pakistani], 1.0% Not Listed; data collected in early 2018). Most worked full-time (87.7%) and nearly half held managerial/supervisory positions (44.7%; Seniority in organization [1-10 scale]: *M* = ­5.04, *SD* = 2.30; Highest level of education: 0.3% No formal qualifications, 8.0% High school/secondary school, 20.8% Some college/A-levels/AS-levels or equivalent, 48.6% Undergraduate degree (BA, BSc, etc.), 18.4% Graduate degree (MA, MSc, MPhil, etc.), 3.8% Doctorate degree (PhD, MD, etc.)). Regarding the number of employees under one’s supervision, 55.0%, 18.5%, 9.9%, 6.0%, 3.3%, 4.6%, 1.7%, 0.3%, and 0.7% of participants reported having 0, 1-5, 5-10, 10-20, 20-50, 50-100, 100-500, 500-1,000, and 1,000 or more employees under their supervision respectively. An additional 79 ineligible respondents were omitted for not matching eligibility criteria (employed full- or part-time at an organization), failing attention checks, not responding to measures used in primary analyses, and/or because the data represented a duplicate submission. Participants were recruited via Prolific to complete a survey about their work experiences. Some did not complete every measure so the number of participants in each analysis varied slightly (lowest *n* = 298). Though standards for power analyses to test models in SEM are not well-established, the proportion of latent factors to manifest variables specified in the hypothesized model and the minimum absolute effect detected among hypothesized structural parameters (*r* = .45) yielded a suggested sample size of 116 (α = .05, 1 - *β* = .80) [1]. Sensitivity analysis for a more conventional test of hierarchical regression (see Table 1, main text) also revealed the sample size was adequate to detect effects within the range of those found (α = .05, 1 - *β* = .80; adequately powered to detect local effects [based on ∆R^2^] of *f* ^2^ ≥ .03).

**Study 1b participants**

Study 1b included 182 participants from Study 1a, recruited for a follow-up survey approximately one year later (data collected in late 2018). Study 1b participants largely mirrored Study 1a participants in terms of demographics (e.g., 51.1% female, 86.3% non-Hispanic white, 3.3% East Asian [e.g., Chinese, Japanese, Korean], 2.2% Black [e.g., Caribbean, African], 2.7% Multiracial, 2.7% Latinx/Hispanic, 1.1% South Asian [e.g., Indian, Pakistani], 1.6% Not Listed). Most worked full-time (84.1%) and nearly half held managerial/supervisory positions (46.2%; Seniority in organization [1-10 scale]: *M* = 4.84, *SD* = 2.18; Highest level of education: 9.3% High school/secondary school, 22.7% Some college/A-levels/AS-levels or equivalent, 47.1% Undergraduate degree (BA, BSc, etc.), 19.2% Graduate degree (MA, MSc, MPhil, etc.), 1.7% Doctorate degree (PhD, MD, etc.)). Regarding the number of employees under one’s supervision, 53.3%, 20.3%, 13.7%, 7.1%, 2.7%, 1.1%, 1.1% and 0.5% of participants reported having 0, 1-5, 5-10, 10-20, 20-50, 50-100, 100-500, and 1,000 or more employees under their supervision respectively. Eight Time 1 respondents were ineligible, having not provided consent to be contacted for the Time 2 survey and/or a valid Prolific identifier at Time 1; additional ineligible respondents, *n* = 112, were omitted following the protocols in Study 1a. The Time 2 survey was an abbreviated version of the original, with the same measures of distinctive and fair treatment, intragroup standing, and belonging. All measures were reliable (all α ≥ .83).

**Study 2 participants**

Participants were 453 individuals employed at organizations in the US and UK (*M_age_* = 37.72, *SD* = 10.39, 53.4% female, 93.6% white/non-Hispanic, 0.4% East Asian [e.g., Chinese, Japanese, Korean], 1.6% Black [e.g., Caribbean, African], 2.4% Multiracial, 0.2% Latinx/Hispanic, 1.6% South Asian [e.g., Indian, Pakistani], 0.2% Not Listed; data collected in 2018). Most worked full-time (75.7%) and over half held supervisory positions (58.3%; Seniority in organization [1-10 scale]: *M* = ­5.07, *SD* = 2.09; Highest level of education: 0.7% No formal qualifications, 12.3% High school/secondary school, 24.2% Some college/A-levels/AS-levels or equivalent, 41.3% Undergraduate degree (BA, BSc, etc.), 18.5% Graduate degree (MA, MSc, MPhil, etc.), 3.0% Doctorate degree (PhD, MD, etc.)). Regarding the number of employees under one’s supervision, 41.7%, 31.3%, 12.1%, 6.4%, 4.9%, 1.1%, 1.5%, 0.2%, and 0.7% of participants reported having 0, 1-5, 5-10, 10-20, 20-50, 50-100, 100-500, 500-1,000, and 1,000 or more employees under their supervision respectively. Additional ineligible respondents were omitted following Study 1 protocols (*n* = 175; including not adhering to manipulation task instructions). Participants were recruited via Prolific to complete an online study about their work experiences. Sensitivity analyses indicated the study was adequately powered (to detect effects of *d* ≥ .24, with covariates in analyses; α = .05, one-tailed, 1 - *β* = .80).

**Study 3 participants**

Participants were 427 individuals employed at organizations across the US and UK, recruited via Prolific (*M_age_* = 38.07, *SD* = 10.45, 63.9% female, 89.7% white/non-Hispanic, 0.9% East Asian [e.g., Chinese, Japanese, Korean], 2.3% Black [e.g., Caribbean, African], 3.5% Multiracial, 0.5% Latinx/Hispanic, 2.6% South Asian [e.g., Indian, Pakistani], 0.5% Not Listed; data collected in 2019). Most participants worked full-time (77.8%), and over half held supervisory positions (58.9%; Seniority in organization [1-10 scale]: *M* = ­5.03, *SD* = 2.10; Highest level of education: 0.2% No formal qualifications, 10.3% High school/secondary school, 23.9% Some college/A-levels/AS-levels or equivalent, 40.7% Undergraduate degree (BA, BSc, etc.), 19.7% Graduate degree (MA, MSc, MPhil, etc.), 5.2% Doctorate degree (PhD, MD, etc.)). Regarding the number of employees under one’s supervision, 41.1%, 29.2%, 12.9%, 8.4%, 4.8%, 1.2%, 1.0%, 0.2%, and 1.2% of participants reported having 0, 1-5, 5-10, 10-20, 20-50, 50-100, 100-500, 500-1,000, and 1,000 or more employees under their supervision respectively. Additional ineligible respondents were omitted using Study 2 protocols (*n* = 143). A priori power analyses suggested a sample size of approximately 400 (based on effect sizes from Study 2 [*d* ~ .25; α = .05, one-tailed, 1 - *β* = .80]) for planned group comparisons.

**Studies 1-3 results**

**S1 Table. Study 1a (work organization) regression analysis, distinctive treatment predicting intragroup standing over other relevant constructs (full statistics).**

|  | *Standardized coefficient* | |  | *Unstandardized coefficient (SE)* | | | | *Confidence Interval* | |
| --- | --- | --- | --- | --- | --- | --- | --- | --- | --- |
|  | Model 1 | Model 2 |  | Model 1 | | Model 2 | | Model 1 | Model 2 |
| Group-serving behavior | .34^***^ | .22^***^ |  | .54^***^ | (.08) | .34^***^ | (.08) | [.39, .69] | [.19, .49] |
| Number of employees under one’s supervision | .13^**^ | .12^**^ |  | .10^**^ | (.04) | .09^**^ | (.03) | [.03, .17] | [.03, .16] |
| Salary (relative to others in org.) | .24^***^ | .18^***^ |  | .13^***^ | (.02) | .10^***^ | (.02) | [.08, .17] | [.05, .14] |
| Procedural fairness | .14^**^ | .15^**^ |  | .20^**^ | (.08) | .23^**^ | (.07) | [.05, .36] | [.08, .37] |
| Fair Treatment | .20^***^ | .13^**^ |  | .37^***^ | (.09) | .24^**^ | (.09) | [.18, .55] | [.06, .41] |
| **Distinctive Treatment** | --- | **.30^***^** |  | --- |  | **.51^***^** | (.08) |  | [.34, .67] |

Total *R^2^* = .55, ∆*R^2^* = .06, *F*(1, 291) = 37.70, *p* < .001.

Local effect size, Cohen’s *f* ^2^ = .13 (small-medium effect).

^**^ *p* ≤ .01; ^***^ *p* ≤ .001.

Study 1a *N* = 302 (employees in companies/organizations).

Regarding multicollinearity: All values for VIF ≤ 1.61, Tolerance ≥ 0.62

Results virtually identical to analyses run with additional covariates (all non-significant; ∆*R^2^* = .06, *F*(1, 265) = 36.73, *p* < .001): level of seniority (self-report), having a managerial/supervisory position, salary (raw/non-relative), years employed in: org.; current position; field/profession overall; age, gender, level of education, employment status (full- vs. part-time).

**S1 Fig. Studies 2 and 3 results.** Experimental effects of distinctive treatment (DT) or fair treatment (FT; each compared to a control condition) on individuals’ sense of standing (Study 2: DT, *d* = .30; Study 3: DT, *d* = .21; FT, *d* = .18, *ns*) and belonging (Study 2: DT, *d* = .10, *ns*; Study 3: DT, *d* = .04, *ns*; FT, *d* = .20) in their work organization (measured on 1-7 scales; *N* = 427). Means represent estimates at the mean of the covariates (as reported in main text). Error bars represent standard errors. These results are equivalent to those illustrated in the main text (Figs 3 and 4).

**Studies 4-6: Additional information**

**Methods**

Study 4 participants were 494 individuals employed at organizations across the US (*M_age_* = 34.66, *SD* = 10.40, 44.8% female, 78.8% white/non-Hispanic, 1.1% American Indian/Alaska Native, 3.5% Asian/Asian American, 6.7% Black/African American, 4.5% Latinx/Hispanic, 5.2% Multiracial, 0.2% Not Listed; data collected in 2016). Most worked full-time (88.3%) and nearly half held supervisory positions (45.6%; Seniority in organization [1-10 scale]: *M* = ­5.84, *SD* = 2.03; Highest level of education: 21.8% High school/GSE, 22.0% Some college/2-year college degree, 42.2% Undergraduate/4-year college degree (BA, BSc, etc.), 14.0% Graduate-level or professional degree (PhD, MD, etc.)). Regarding the number of employees under one’s supervision, 54.4%, 20.4%, 12.7%, 7.3%, 3.2%, 1.1%, 0.4%, and 0.4% of participants reported having 0, 1-5, 5-10, 10-20, 20-50, 50-100, 100-500, and 1,000 or more employees under their supervision respectively. Additional ineligible respondents, *n* = 9, were omitted for not matching eligibility criteria [employed full- or part-time at an organization]; note that these criteria were made explicit in the study’s recruitment posting). Participants were recruited via Amazon Mechanical Turk and asked to complete an online survey about their work experiences. Some did not complete every measure, so the number of participants in each analysis varied slightly (lowest *n* = 460). Power analyses based on the proportion of latent factors to manifest variables in the hypothesized model and the minimum absolute effect detected among hypothesized structural parameters (*r* = .30; α = .05, 1 - *β* = .80) yielded a suggested sample size of 170 [1]. Sensitivity analysis for a more conventional test of hierarchical regression (see S4 Table) also revealed the sample size was adequate (at lowest *n*, α = .05, 1 - *β* = .80; adequately powered to detect local effect sizes of *f* ^2^ ≥ .02).

Study 5 participants were 190 undergraduates from a large US public university (*M*_age_ = 19.23, *SD* = 1.30, 72.9% female, 0.5% American Indian/Alaska Native, 40.5% Asian/Asian American, 3.2% Black/African American, 23.8% Latinx/Hispanic, 9.7% Multiracial, 21.1% white/non-Hispanic, 1.1% Not Listed; 3.8% international students [vs. not], 2.2% transfer students [vs. not]; data collected in 2016). Most participants were in their first year at the university (53.5%; another 33.0%, 7.0%, 5.4% and 1.1% were in their second, third, fourth and fifth years at the university, respectively). Additional respondents, *n* = 18, were omitted for not meeting eligibility criteria [aged 18+ currently enrolled at the university] and/or not completing measures used in primary analyses). Participants were recruited through a participant research pool to complete an online survey about their everyday experiences with other undergraduates at the university. Study 6 participants were 322 US-born Asian/Asian American and Latinx/Hispanic students from a large US public university (*M*_age_ = 20.70, *SD* = 2.13, 73.0% female; 43.8% Asian/Asian American, 56.2% Latinx/Hispanic; additional respondents, *n* = 19, were omitted for not meeting eligibility criteria [US-born minority individuals in one of the aforementioned racial/ethnic groups] and/or not completing measures used in primary analyses; data collected in 2014-15). Participants were recruited via email from the university’s registrar’s office. They completed an online survey about their everyday experiences with other members of their pan-ethnic group (Asian/Asian American or Latinx/Hispanic; note: participants were also part of a longitudinal study examining processes related to discrimination and health; by comparison, distinctive treatment was only assessed at one time point, and prior descriptions of the data [2] do not involve any analyses of distinctive or fair treatment). Effects found in Study 4 (i.e., the minimum absolute effect among hypothesized structural parameters, *r* = .30 [α = .05, 1 - *β* = .80], and given the proportion of latent factors to manifest variables in the hypothesized model [1]) provided a basis for estimating appropriate sample sizes for Studies 5-6 (approx. 170, though we were able to recruit a larger [voluntary] sample for Study 6, partly in anticipation of conducting preliminary multiple groups analyses).

**Materials**

Studies 4-6 measured fair and distinctive treatment, intragroup standing and belonging, group identification, perceived control, and mental health using nearly identical items. All measures in all studies were reliable (α ≥ .76). They were modified as necessary to reflect the relevant group context (e.g., undergraduate community, racial/ethnic minority group). Items for each study are reported in a subsequent section of *SI*. One intragroup standing item (“...admired”) was not measured in Study 6. As in earlier studies, we also tested whether distinctive treatment predicted intragroup standing over and above individuals’ own group-serving behaviors, and other potential indicators of one’s standing.

**Distinctive and fair treatment, intragroup standing and belonging**

Distinctive and fair treatment were measured using the same items as in Study 1. Perceived intragroup standing was also measured using the same items as in Study 1, with one additional item (“...seen as a leader within this [group; e.g., ‘organization’ in Study 4]”). Perceived intragroup belonging was also measured using the same items as Study 1 and the additional item used in Studies 2-3.

**Group identification**

Four items from Leach et al. [3] measured individuals’ group identification. For instance, in Study 4: (i) “Being a part of this organization is an important part of how I see myself,” (ii) “I am glad to be a part of this organization,” (iii) “Being part of this organization gives me a good feeling,” (iv) “The fact that I am part of this organization is an important part of my identity” (1 *strongly disagree* – 7 *strongly agree*).

**Perceived control**

Six items from Lachman et al. [4,5] measured individuals’ perceived control over life (e.g., “Whether or not I am able to get what I want is in my own hands,” “I have little control over the things that happen to me” [reverse coded]; 1 *strongly disagree* – 7 *strongly agree*).

**Mental health**

Mental health was assessed in two ways: (i) trait-anxiety, using six items from Spielberger [6] (e.g., “I worry about things that don’t really matter,” 1 *never* - 5 *very often*), (ii) depressive symptoms, using the 10 item CES-D (Boston Form) from Kohout et al. [7] (e.g., “I felt depressed,” 0 *never* - 3 *very often*).

**Additional indicators of standing**

As in Study 1, to more rigorously test the predictive strength of distinctive treatment we measured other potential indicators of standing (e.g., in Study 4, holding a managerial/supervisory position; for complete lists, see S4-S6 Tables).

**Results**

Summary statistics and bivariate correlations are in S2 and S3 Tables.

**Empirically distinguishing key constructs**

As in Study 1 (using the same protocols), we tested whether distinctive and fair treatment represented independent constructs. In each study, eigenvalues and scree plots indicated clear two-factor solutions with all items loading onto their appropriate factors without substantial cross-loadings. We also tested whether distinctive treatment and intragroup standing represented independent constructs. Results again demonstrated clear two-factor solutions with all items loading onto their appropriate factors without substantial cross-loadings. See Table 1 (main text) for more detail.

**S2 Table. Study 4 (work organization) means, standard deviations and bivariate correlations.**

| Variable | *Mean* | *SD* | 1 | 2 | 3 | 4 | 5 | 6 | 7 |
| --- | --- | --- | --- | --- | --- | --- | --- | --- | --- |
| 1. Distinctive Treatment | 3.58 ^a^ | 0.82 ^d^ | ----- |  |  |  |  |  |  |
| 2. Fair Treatment | 3.80 ^a^ | 0.75 ^d^ | .44 | ----- |  |  |  |  |  |
| 3. Intragroup Standing | 4.77 ^b^ | 1.26 ^e^ | .64 | .47 | ----- |  |  |  |  |
| 4. Intragroup Belonging | 5.56 ^b^ | 1.03 ^e^ | .46 | .66 | .60 | ----- |  |  |  |
| 5. Group Identity | 4.65 ^b^ | 1.40 | .36 | .52 | .50 | .45 | ----- |  |  |
| 6. Personal Control | 4.99 ^b^ | 1.16 | .32 | .45 | .39 | .47 | .24 | ----- |  |
| 7. Anxiety | 2.35 ^a^ | 0.81 | -.30 | -.48 | -.43 | -.52 | -.29 | -.72 | ----- |
| 8. Depressive Symptoms | 0.72 ^c^ | 0.60 | -.19 | -.41 | -.29 | -.43 | -.20 | -.67 | .79 |

^a^ 1-5 scale, ^b^ 1-7 scale, ^c^ 0-3 scale; all correlations significant at *p* ≤ .001

^d^ Means for distinctive- and fair treatment are significantly different, *t*(468) = 5.75, *p* < .001

^e^ Means for intragroup standing and belonging are significantly different, *t*(471) = 16.44, *p* < .001

**S3 Table. Study 5 (student community) and Study 6 (racial/ethnic minority groups) means, standard deviations and bivariate correlations.**

| Variable | *Mean* | *SD* | 1 | 2 | 3 | 4 | 5 | 6 | 7 | 8 |  | *Mean* | *SD* |
| --- | --- | --- | --- | --- | --- | --- | --- | --- | --- | --- | --- | --- | --- |
| 1. Distinctive Treatment ^a^ | 3.28 ^d^ | 0.72 | -- | .38 | .61 | .45 | .29 | .24 | -.22 | -.07 ^i^ |  | 3.54 ^d^ | 0.76 |
| 2. Fair Treatment ^a^ | 3.72 ^d^ | 0.66 | .53 | -- | .37 | .59 | .35 | .28 | -.23 | -.15 ^f^ |  | 3.75 ^d^ | 0.58 |
| 3. Intragroup Standing ^b^ | 3.92 ^e^ | 1.22 | .56 | .42 | -- | .51 | .33 | .31 | -.35 | -.14 ^f^ |  | 4.81 ^e^ | 1.29 |
| 4. Intragroup Belonging ^b^ | 5.40 ^e^ | 0.95 | .46 | .61 | .49 | -- | .36 | .34 | -.37 | -.31 |  | 5.60 ^e^ | 0.89 |
| 5. Group Identity ^b^ | 5.45 | 1.13 | .33 | .44 | .37 | .46 | -- | .13 ^g^ | -.11 ^h^ | -.03 ^i^ |  | 5.69 | 1.13 |
| 6. Personal Control ^b^ | 4.74 | 0.87 | .28 | .29 | .34 | .31 | .28 | -- | -.69 | -.56 |  | 4.94 | 0.93 |
| 7. Anxiety ^a^ | 2.77 | 0.61 | -.25 | -.39 | -.44 | -.46 | -.33 | -.48 | -- | .64 |  | 2.64 | 0.66 |
| 8. Depressive Symptoms ^c^ | 0.91 | 0.49 | -.23 | -.39 | -.34 | -.43 | -.32 | -.47 | .68 | -- |  | 0.62 | 0.49 |

Study 5/6 data are below/above the diagonal.

^a^ 1-5 scale, ^b^ 1-7 scale, ^c^ 0-3 scale; correlations significant at *p* ≤ .001 except as noted: ^f^ *p* ≤ .01; ^g^ *p* ≤ .05; ^h^ *p* ≤ .10; ^i^ *ns*.

^d^ Means for distinctive- and fair treatment are significantly different, *t*’s ≥ 5.11, *p*’s < .001.

^e^ Means for intragroup standing and belonging are significantly different, *t*’s ≥ 12.54, *p*’s < .001.

**Testing the robustness of distinctive treatment over other indicators of one’s standing**

We also assessed whether distinctive treatment predicted individuals’ perceived intragroup standing over and above other relevant indicators. As in Study 1, we ran hierarchical regression analyses with other indicators in the first step, and distinctive treatment in a second step. Replicating Study 1 findings, results showed these other indicators predicted individuals’ sense of standing. Yet when distinctive treatment was added to the model it emerged as one of the strongest predictors. This held true across all three studies / group contexts (S4-S6 Tables).

**S4 Table. Study 4 (work organization) regression analyses, with distinctive treatment predicting intragroup standing over and above several control variables (full statistics).**

|  | *Standardized coefficient* | |  | *Unstandardized coefficient (SE)* | | | | *Confidence Interval* | |
| --- | --- | --- | --- | --- | --- | --- | --- | --- | --- |
|  | Model 1 | Model 2 |  | Model 1 | | Model 2 | | Model 1 | Model 2 |
| Group-serving behavior | .36^***^ | .21^***^ |  | .50^***^ | (.06) | .21^***^ | (.06) | [.39, .61] | [.18, .41] |
| Fair treatment | .30^***^ | .20^***^ |  | .50^***^ | (.07) | .20^***^ | (.06) | [.38, .63] | [.21, .46] |
| Number of employees under one’s supervision | .10^**^ | .08^*^ |  | .09^**^ | (.04) | .08^*^ | (.03) | [.02, .16] | [.01, .14] |
| Salary (relative to others in org.) | .15^***^ | .12^***^ |  | .01^***^ | (.002) | .12^***^ | (.002) | [.004, .01] | [.002, .01] |
| Time spent with others of lower standing in org.^a^ | .15^***^ | .09^**^ |  | .17^***^ | (.04) | .09^**^ | (.04) | [.09, .25] | [.03, .19] |
| **Distinctive Treatment** | --- | **.36^***^** |  | --- |  | **.36^***^** | (.07) |  | [.43, .69] |

Total *R*^2^ = .52, ∆*R*^2^ = .07, *F*(1, 453) = 70.71, *p* < .001.

Local effect size, Cohen’s *f* ^2^ = .16 (medium effect).

^*^ *p* ≤ .05; ^**^ *p* ≤ .01; ^***^ *p* ≤ .001.

Study 4 *N* = 494 (employees in companies/organizations).

^a^ Because opportunities to make downward comparisons might elevate one’s own relative sense of standing [8], we asked how much time participants typically spent interacting with employees who have lower standing than them (1 *none of my time* – 5 *all of my time*).

Regarding multicollinearity: All values for VIF ≤ 1.75, Tolerance ≥ 0.57

Results virtually identical to analyses run with additional covariates (all non-significant; ∆*R^2^* = .07, *F*(1, 443) = 64.23, *p* < .001): age, gender, level of education, salary (raw/non-relative), years employed in org., employment status (full-time vs. part-time).

**S5 Table. Study 5 (student community) regression analyses, distinctive treatment predicting intragroup standing over and above control variables (full statistics).**

|  | *Standardized coefficient* | |  | *Unstandardized coefficient (SE)* | | | | *Confidence Interval* | |
| --- | --- | --- | --- | --- | --- | --- | --- | --- | --- |
|  | Model 1 | Model 2 |  | Model 1 | | Model 2 | | Model 1 | Model 2 |
| Group-serving behavior | .36^***^ | .26^***^ |  | .52^***^ | (.09) | .37^***^ | (.09) | [.35, .69] | [.20, .54] |
| Fair Treatment | .38^***^ | .21^**^ |  | .70^***^ | (.11) | .39^**^ | (.12) | [.48, .92] | [.15, .63] |
| Gender ^a^ | .15^**^ | .18^***^ |  | .43^**^ | (.17) | .50^***^ | (.16) | [.10, .76] | [.20, .81] |
| International student ^b^ | -.21^***^ | -.19^***^ |  | -1.34^***^ | (.39) | -1.21^***^ | (.36) | [-2.10, -.58] | [-1.93, -.50] |
| **Distinctive Treatment** | --- | **.36^***^** |  | --- |  | **.62^***^** | (.12) |  | [.38, .85] |

Total *R*^2^ = .47, ∆*R*^2^ = .08, *F*(1, 174) = 26.67, *p* < .001

Local effect size, Cohen’s *f* ^2^ = .15 (medium effect).

^**^ *p* ≤ .01; ^***^ *p* ≤ .001.

Study 5 *N* = 190 (undergraduate students).

^a^ 0 = *Woman*, 1 = *Man*.

^b^ 0 = *No*, 1 = *Yes*.

Regarding multicollinearity: All values for VIF ≤ 1.59, Tolerance ≥ 0.63

Results virtually identical to analyses run with additional covariates (all non-significant; ∆*R^2^* = .08, *F*(1, 156) = 21.63, *p* < .001): year in school, household income, being a transfer student.

**S6 Table. Study 6 (racial/ethnic minority groups) regression analyses, distinctive treatment predicting intragroup standing over and above several control variables (full statistics).**

|  | *Standardized coefficient* | |  | *Unstandardized coefficient (SE)* | | | | *Confidence Interval* | |
| --- | --- | --- | --- | --- | --- | --- | --- | --- | --- |
|  | Model 1 | Model 2 |  | Model 1 | | Model 2 | | Model 1 | Model 2 |
| Group-serving behavior | .24^***^ | .10^*^ |  | .27^***^ | (.06) | .12^*^ | (.10) | [.15, .39] | [.01, .22] |
| Fair Treatment | .31^***^ | .14^**^ |  | .68^***^ | (.12) | .32^**^ | (.14) | [.45, .91] | [.11, .52] |
| **Distinctive Treatment** | --- | **.52^***^** |  | --- |  | **.89^***^** | (.52) |  | [.72, 1.05] |

Total *R*^2^ = .40, ∆*R*^2^ = .22, *F*(1, 318) = 114.67, *p* < .001.

Local effect size, Cohen’s *f* ^2^ = .36 (large effect).

^***^ *p* ≤ .001; ^**^ *p* ≤ .01; ^*^ *p* ≤ .05

Study 6 *N* = 322 (racial/ethnic minority group members).

Regarding multicollinearity: All values for VIF ≤ 1.26, Tolerance ≥ 0.79

Results virtually identical to analyses run with additional covariates (all non-significant; ∆*R^2^* = .21, *F*(1, 312) = 112.36, *p* < .001): age, gender, household income.

**Primary analyses**

We tested the strength of hypothesized processes using SEM in EQS [9]. Latent factors were specified for each construct (distinctive treatment, fair treatment, intragroup standing, and belonging using their respective items as indicators, identity, personal control, and mental health using respective subscale composites as indicators; centrality/satisfaction, mastery/constraints, anxiety/depressive symptoms, enabling unbiased parameter estimates without an overly complex measurement model). Data were analyzed using robust maximum likelihood estimation [10].

Overall, in each study, results showed that the model fit well, Study 4: SB χ^2^ (220) = 453.1, *p* < .001, CFI = .96, RMSEA = .05 [.042, .054]; Study 5: SB χ^2^ (220) = 289.3, *p* = .001, CFI = .97, RMSEA = .04 [.027, .053], Study 6: SB χ^2^ (199) = 341.2, *p* < .001, CFI = .95, RMSEA = .05 [.039, .056]. Moreover, in each study, path coefficients supported each prediction. Individuals’ perceived standing was strongly predicted by experiences with distinctive treatment, while fair treatment was a relatively weak predictor. By comparison, individuals’ sense of belonging was strongly predicted by experiences with fair treatment, while distinctive treatment was a relatively weak predictor. Individuals’ sense of standing and belonging subsequently predicted stronger group identification, which in turn predicted a greater sense of control over life and better mental health (Fig 4, main text). Thus, results indicated that within a variety of important real-world groups distinctive and fair treatment played unique roles in explaining individuals’ sense of standing and belonging within those groups. Moreover, a strong sense of standing and belonging had positive implications for mental health, explained through identity-based processes.

As in Study 1, to further assess whether distinctive and fair treatment played unique roles in explaining individuals’ intragroup standing and belonging, we directly compared the magnitude of their effects (*r*). As expected, in Studies 4/5/6 distinctive treatment was a stronger predictor of intragroup standing than fair treatment, *z* = 4.51/2.78/5.30, *p* ≤ .01. By comparison, fair treatment was a stronger predictor of belonging than distinctive treatment, *z* = 7.22/3.09/3.10, *p* ≤ .001. These results further supported the idea that distinctive and fair treatment played unique roles in explaining individuals’ sense of standing and belonging, evinced across a multitude of important real-world groups.

**Testing an alternative model**

To further assess the importance of distinctive treatment as a separate form of group-based treatment, we specified an alternative model in which distinctive treatment was absent. Replicating Study 1 findings, results showed this alternative model fit reasonably well and fair treatment predicted perceived standing and belonging. However, this model accounted for relatively little variance on intragroup standing, Study 4/5/6 *R*^2^_intragroup standing_ = .28/.19/.17. By comparison, the hypothesized model accounted for approximately twice that, *R*^2^_intragroup standing_ = .52/.37/.45, ∆R^2^ = .24/.18/.28, Cohen’s *f* ^2^ = .50/.29/.51 (medium to large effect). Thus, across these different groups, results indicated that individuals had meaningful experiences of distinctive treatment, and accounting for them provided a more comprehensive basis for understanding their relational self-concept than fair treatment alone (with implications for their mental health).

**Study measures and stimuli**

Across all (non-experimental) studies, measures were ordered in surveys to generally run counter to the hypothesized direction of processes. For all constructs measured using multiple items, these items were averaged to form a composite, with higher values representing greater frequency or levels of that construct (e.g., higher frequency of experiencing distinctive treatment, greater levels of intragroup standing, group identification, mental health).

**Study 1**

**Distinctive treatment**

Thinking about the other employees you interact with in this organization (face-to-face, via phone, email, etc.), how often do they...?: ask you for advice; look to you for guidance when they have a question or problem; ask you to share your opinions and ideas about things; ask you for help because of certain knowledge, skills or perspectives you have (1 *never* – 5 *very often*).

**Fair treatment**

Thinking about the other employees you interact with in this organization (face-to-face, via phone, email, etc.), how often do they...?: treat you fairly; show care for your well-being; treat you with openness and honesty; take your needs into consideration (1 *never* – 5 *very often*).

**Intragroup standing**

Within this organization (among employees), I feel that I am...: looked up to; admired; seen as a role model for others in the organization; held in high regard (1 *strongly disagree* – 7 *strongly agree*).

**Intragroup belonging**

Within this organization (among employees), I feel that I am...: accepted for who I am; considered a nice person to have around; well-liked as a person (1 *strongly disagree* – 7 *strongly agree*).

**Control variables**

Group-serving behavior: Within this organization, how often do you...?: do things over and above what is expected of you to help improve the organization; go out of your way to help supervisors, managers or others of high standing in the organization; spend time outside of work thinking about new ways to help improve the functioning of the organization; devote more of your time / energy to work than is expected of you (1 *never* – 5 *very often*). Standing indicators: If you had to guess, how many employees at this organization make more money than you? (10 *0%* – 0 *100%*); What is your current salary (before taxes)? (1 *Up to £15,000* – 12 *More than £150,000*); Within the company/organization you work for, what is your current level of seniority (i.e., relative standing of your position)? (1 *Very Junior/Low Level Position* – 10 *Very Senior/High Level Position*); How many employees are directly under your supervision? (0 *0 people* – 8 *Over 1,000 people*); More generally, do you hold a managerial or supervisory position in this organisation? (0 *No* – 1 *Yes*); How long have you worked...?: at this organization; in the particular position you currently hold in the organization; in this field/profession, more generally (1 *Less than 1 year* – 9 *More than 20 years*); What is your highest achieved education level? (1 *No Formal Qualifications* – 6 *Doctorate Degree (PhD, MD, etc.)*); Are you currently employed? (1 *Yes, part-time* – 2 *Yes, full-time* [respondents ineligible for study if selecting 0 *No, I am currently not employed*]). Procedural fairness: Overall, regarding the procedures/policies your organization uses, to what extent…?: are you able to express your views during these procedures; can you influence the decisions that are made via these procedures; are these procedures applied consistently; are these procedures free of bias; are these procedures based on accurate information; are you able to appeal the decisions arrived at by these procedures; do these procedures uphold ethical and moral standards; (1 *to a small extent* – 5 *to a large extent*).

**Study 2**

**Distinctive treatment condition / manipulation**

Here, we would like you take a few minutes to think about some of your experiences with the people you work with – this could include supervisors or managers, co-workers, subordinates, etc. In particular, please think about some past situations in which another employee, or group of employees... - came to you for ideas, advice or guidance on how to address some work-related issue, or… - sought you out because of a particular skill, type of knowledge, or area of expertise that you possess (that could help address a challenging issue at work, improve the quality of a work project, etc.). Below, please describe two of these types of past situations. For each, please type at least 4-5 sentences describing what happened. You might also include some details about who was involved (e.g., co-workers, supervisors/managers) and how often this type of situation occurs.

Situation #1 [open-ended text box]

Situation #2 [open-ended text box]

**Control condition**

Here, we would like you to take a few minutes to think about the route you take to get to work, and the route you take when leaving work (e.g., to get home at the end of the day). This might include the roads/paths you take, the distance travelled, the amount of time it takes, the general surroundings along the route, etc. For each, please type at least 4-5 sentences describing the route.

1. Route to Work [open-ended text box]

2. Route from Work [open-ended text box]

**Intragroup standing**

Within this organization (among other employees), I feel that I am...: looked up to; admired; seen as a role model for others in the organization; held in high regard (1 *Strongly Disagree* – 7 *strongly agree*).

**Intragroup belonging**

Within this organization (among other employees), I feel that I am...: accepted for who I am; enjoyed for my company; well-liked as a person (1 *strongly disagree* – 7 *strongly agree*).

**Control variables**

Within the company/organization you work for, what is your current level of seniority (relative standing of your position)? (1 *Very Junior/Low Level Position* – 10 *Very Senior/High Level Position*); How many employees are directly under your supervision? (0 *0 people* – 8 *Over 1,000 people*); What is your highest achieved education level? (1 *No Formal Qualifications* – 6 *Doctorate Degree (PhD, MD, etc.)*); Are you currently employed? (1 *Yes, part-time* – 2 *Yes, full-time*).

**Manipulation check and follow-up**

Earlier in the survey we asked you to describe a couple of situations (in 4-5 sentences) related to your work. What type of situations were you asked to describe? (0 *The route I take to and from work*, 1 *Two past situations in which other employees called on me to provide advice, guidance, skills, knowledge, etc.)*. When you were asked to describe two past situations where another employee (or group of employees) called upon you to provide advice, guidance, skills, knowledge, etc., how difficult was it to think of two such instances or situations to describe? (for distinctive treatment condition; 1 *not at all difficult* – 7 *very difficult*). When you were asked to think of the route you take to work, and from work, how difficult was it to think of these two routes to describe? (for control condition; 1 *not at all difficult* – 7 *very difficult*).

**Study 3**

**Distinctive treatment condition / manipulation**

Here, we would like you take a few minutes to think about some of your experiences with the people you work with – this could include supervisors or managers, co-workers, subordinates, etc. In particular, please think about some past situations in which another employee, or group of employees... - came to you for ideas, advice or guidance on how to address some work-related issue, or… - sought you out because of a particular skill, type of knowledge, or area of expertise that you possess (that could help address a challenging issue at work, improve the quality of a work project, etc.). Below, please describe two of these types of past situations. For each, please type at least 4-5 sentences describing what happened. You might also include some details about who was involved (e.g., co-workers, supervisors/managers) and where you were when this happened.

Situation #1 [open-ended text box]

Situation #2 [open-ended text box]

**Fair treatment condition / manipulation**

Here, we would like you take a few minutes to think about some of your experiences with the people you work with – this could include supervisors or managers, co-workers, subordinates, etc. In particular, please think about some past situations in which another employee, or group of employees... - treated you in a way that was generally fair, honest or considerate when dealing with a work-related issue, or… - showed care for your well-being, or took your needs into consideration (when addressing a work-related matter, working on a task or project at work, etc.). Below, please describe two of these types of past situations. For each, please type at least 4-5 sentences describing what happened. You might also include some details about who was involved (e.g., co-workers, supervisors/managers) and where you were when this happened.

Situation #1 [open-ended text box]

Situation #2 [open-ended text box]

**Control condition**

Here, we would like you to take a few minutes to think about the route you take to get to work, and the route you take when leaving work (e.g., to get home at the end of the day). This might include the roads/paths you take, the distance travelled, the amount of time it takes, the general surroundings along the route, etc. For each, please type at least 4-5 sentences describing the route.

1. Route to Work [open-ended text box]

2. Route from Work [open-ended text box]

**Intragroup standing**

Note that the order of the intragroup standing and belonging measures was randomized to avoid any influence of their temporal proximity to the manipulation.

Within this organization (among other employees), I feel that I am...: looked up to; admired; seen as a role model for others in the organization; held in high regard (1 *Strongly Disagree* – 7 *strongly agree*).

**Intragroup belonging**

Within this organization (among other employees), I feel that I am...: accepted for who I am; enjoyed for my company; well-liked as a person (1 *Strongly Disagree* – 7 *Strongly Agree*).

**Control variables**

Within the company/organization you work for, what is your current level of seniority (relative standing of your position)? (1 *Very Junior/Low Level Position* – 10 *Very Senior/High Level Position*); How many employees are directly under your supervision? (0 *0 people* – 8 *Over 1,000 people*); If you had to guess, how many employees at this organization make more money than you? (10 *0%* – 0 *100%*); What is your highest achieved education level? (1 *No Formal Qualifications* – 6 *Doctorate Degree (PhD, MD, etc.)*); What is your employment status? (1 *Part-time* – 2 *Full-time*).

**Manipulation check and follow-up**

Earlier in the survey we asked you to describe a couple of situations (4-5 sentences) related to your work. What type of situation were you asked to describe? (0 *The route I take to and from work*, 1 *Situations in which other employees treated me in a way that was fair, honest, caring or considerate*, 2 *Situations in which other employees called on me to provide advice, guidance, skills, knowledge, etc.)*. When you were asked to describe two past situations where another employee (or group of employees) called upon you to provide advice, guidance, skills, knowledge, etc., how difficult was it to think of two such instances or situations to describe? (for distinctive treatment condition; 1 *not at all difficult* – 7 *very difficult*). When you were asked to describe two past situations where another employee (or group of employees) treated you in a way that was fair, honest, caring or considerate, how difficult was it to think of two such instances or situations to describe? (for fair treatment condition; 1 *not at all difficult* – 7 *very difficult*). When you were asked to think of the route you take to work, and from work, how difficult was it to think of these two routes to describe? (for control condition; 1 *not at all difficult* – 7 *very difficult*).

**Study 4**

**Distinctive treatment**

When you interact with other employees in this organization, how often do they...?: ask you for advice; look to you for guidance when they have a question or problem; ask you to share your opinions and ideas about things; ask you for help because of certain knowledge, skills or perspectives you have (1 *never* – 5 *very often*).

**Fair treatment**

When you interact with other employees in this organization, how often do they...?: treat you fairly; show care for your well-being; treat you with openness and honesty; take your needs into consideration (1 *never* – 5 *very often*).

**Intragroup standing**

Within this organization (among employees), I feel that I am...: looked up to; admired by others; seen as a role model for others in the organization; seen as a leader within this organization; held in high regard (1 *strongly disagree* – 7 *strongly agree*).

**Intragroup belonging**

Within this organization (among employees), I feel that I am...: accepted for who I am; considered a nice person to have around; enjoyed for my company; well-liked as a person (1 *strongly disagree* – 7 *strongly agree*).

**Group identification**

Please think about the following organization [self-reported name of org. piped into text]: Being a part of this organization is an important part of how I see myself; I am glad to be a part of this organization; being part of this organization gives me a good feeling; the fact that I am part of this organization is an important part of my identity (1 *strongly disagree* – 7 *strongly agree*).

**Personal control**

These statements have to do with your general feelings about life and your future. Please indicate how much you agree or disagree with the following statements: I can do just about anything I set my mind to; when I really want to do something, I usually find a way to succeed at it; whether or not I am able to get what I want is in my own hands; I often feel helpless in dealing with the problems of life; I have little control over the things that happen to me; there are many things that interfere with what I want to do (last 3 items reverse-scored; 1 *strongly disagree* – 7 *strongly agree*).

**Mental health**

Trait-anxiety: In general, how often do these statements apply to you: I worry about things that really don't matter; I lack self-confidence; I feel inadequate; I feel pleasant; I feel satisfied with myself; I am happy (last 3 items reverse-scored; 1 *never* – 5 *very often*). Depressive symptoms: In the past week...: I felt depressed; I could not "get going;" my sleep was restless; I felt that people disliked me; I felt lonely; people were unfriendly; I felt everything I did was an effort; I felt sad; I was happy; I enjoyed life (last 2 items reverse-scored; 0 *never/rarely* – 3 *very often*).

**Control variables**

Group-serving behavior: Please think about the following organization [self-reported name of org. piped into text] How often do you...?: do things over and above what is expected of you to improve the organization; think about what you can do to make the organization stronger; talk with others about ways to improve the reputation or functioning of your organization (1 *never* – 5 *very often*). Standing indicators: If you had to guess, how many employees at this organization make more money than you? (100 *Almost no one* – 0 *Almost everyone*); What is your current salary (before taxes)? (1 *Under $20,000* – 7 *$150,000 or more*); Think of this ladder (right) as representing where all the employees at your organization stand – employees at the top of the ladder have the best positions in the organization (e.g., make the most money, or have the most "prestigious" job titles), employees at the bottom have the worst positions in the organization (e.g., make the least money, or have the least "prestigious" job titles) – Where would you place yourself on this ladder, compared to all the other employees at this organization? (1 *[Lowest Standing/Rung on Ladder]* – 10 *[Highest Standing/Rung on Ladder]*); How many employees are directly under your supervision? (0 *0 people* – 8 *Over 1,000 people*); Do you currently hold a managerial or supervisory position in this organisation? (0 *No* – 1 *Yes*); In a typical week, how much of your time is spent interacting with employees who generally have 'lower standing' in the organization than you? (1 *None of my time* – 5 *Almost all of my time*); How long have you been employed at this organization (1 *Less than a year* – 4 *More than 10 years*); What is the highest level of education you have completed? (1 *Less than high school* – 5 *Graduate college or professional degree (JD, MD, DMD)*); Are you currently employed (part-time, full-time)? (1 *Yes, part-time* – 2 *Yes, full-time* [respondents ineligible for study if selecting 0 *No, I am currently not employed*]).

**Study 5**

**Distinctive treatment**

When you interact with other [name of university] undergraduate students, how often do they...?: ask you for advice; look to you for guidance when they have a question or problem; ask you to share your opinions and ideas about things; ask you for help because of certain knowledge, skills or perspectives you have (1 *never* – 5 *very often*).

**Fair treatment**

When you interact with other [name of university] undergraduate students, how often do they...?: treat you fairly; show care for your well-being; treat you with openness and honesty; take your needs into consideration (1 *never* – 5 *very often*).

**Intragroup standing**

Among [name of university] undergraduate students, I feel that I am...: looked up to; admired; seen as a role model for other [university] students; seen as a leader among [university] undergraduates; held in high regard (1 *strongly disagree* – 7 *strongly agree*).

**Intragroup belonging**

When I am around other [name of university] undergraduate students, I generally feel that I am...: accepted for who I am; considered a nice person to have around; enjoyed for my company; well-liked as a person (1 *strongly disagree* – 7 *strongly agree*).

**Group identification**

Please indicate how much you agree or disagree with the following statements about the [name of university] undergraduate student body: Being a part of the [university] undergraduate student body is an important part of how I see myself; I am glad to be a [university] student; being a [university] undergraduate student gives me a good feeling; the fact that I am a [university] student is an important part of my identity (1 *strongly disagree* – 7 *strongly agree*).

**Personal control**

These statements have to do with your general feelings about life and your future. Please indicate how much you agree or disagree with the following statements: I can do just about anything I set my mind to; when I really want to do something, I usually find a way to succeed at it; whether or not I am able to get what I want is in my own hands; I often feel helpless in dealing with the problems of life; I have little control over the things that happen to me; there are many things that interfere with what I want to do (last 3 items reverse-scored; 1 *strongly disagree* – 7 *strongly agree*).

**Mental health**

Trait-anxiety: In general, how often do these statements apply to you: I worry about things that really don't matter; I lack self-confidence; I feel inadequate; I feel pleasant; I feel satisfied with myself; I am happy (last 3 items reverse-scored; 1 *never* – 5 *very often*). Depressive symptoms: In the past week...: I felt depressed; I could not "get going;" my sleep was restless; I felt that people disliked me; I felt lonely; people were unfriendly; I felt everything I did was an effort; I felt sad; I was happy; I enjoyed life (last 2 items reverse-scored; 0 *never/rarely* – 3 *very often*).

**Control variables**

Group-serving behavior: How often do you...?: do things over and above what is expected of you to help [name of university] undergraduate students; think about what you can do to make [university] or the [university] undergraduate student body stronger; talk with others about ways to improve the reputation or functioning of the [university] undergraduate student body (1 *never* – 5 *very often*). Standing indicators: What year are you in at [university]? (1 *First year/Freshman* – 5 *5^th^ year or higher*). What is your family's annual household income? If you are not sure, please give your best estimate (1 *Under $20,000* – 7 *$150,000 or more*); Are you an international student? (0 *No* – 1 *Yes*); Are you a transfer student? (0 *No* – 1 *Yes*).

**Study 6**

Where relevant, items were preceded by the following text: At the beginning of this survey, you stated that you most highly identify as [self-selected race/ethnicity]. Please think about this racial/ethnic group when responding to the following statements. Participants’ selected racial/ethnic group (Asian / Asian American, Latino(a) / Hispanic) was piped into the text of relevant items, designated below as ‘[race/ethnicity].’

**Distinctive treatment**

How often do people in your racial/ethnic group...?: ask you for advice; look to you for support or guidance when they have a problem; ask you to share your opinions and ideas about things; rely on you for help because of certain talents, skills or knowledge you have (1 *never* – 5 *very often*).

**Fair treatment**

When you interact with people in your racial/ethnic group, how often do you feel that...?: they treat you fairly; they care about your well-being; they are honest with you; they are considerate or sensitive to your needs (1 *never* – 5 *very often*).

**Intragroup standing**

Most of the time, I feel that people in my racial/ethnic group...: look up to me; see me as a role model for others in my racial/ethnic group; see me as a leader in my racial/ethnic group; hold me in high regard (1 *strongly disagree* – 7 *strongly agree*).

**Intragroup belonging**

Most of the time, I feel that people in my racial/ethnic group...: accept me for who I am; consider me to be a nice person to have around; enjoy my company; like me as a person (1 *strongly disagree* – 7 *strongly agree*).

**Group identification**

Being [race/ethnicity] is an important part of how I see myself; I am glad to be [race/ethnicity]; being [race/ethnicity] gives me a good feeling; the fact that I am [race/ethnicity] is an important part of my identity (1 *strongly disagree* – 7 *strongly agree*).

**Personal control**

These statements have to do with your general feelings about life and your future. Please indicate how much you agree or disagree with the following statements: I can do just about anything I set my mind to; when I really want to do something, I usually find a way to succeed at it; whether or not I am able to get what I want is in my own hands; I often feel helpless in dealing with the problems of life; I have little control over the things that happen to me; there are many things that interfere with what I want to do (last 3 items reverse-scored; 1 *strongly disagree* – 7 *strongly agree*).

**Mental health**

Trait-anxiety: In general, how often do these statements apply to you: I worry about things that really don't matter; I lack self-confidence; I feel inadequate; I feel pleasant; I feel satisfied with myself; I am happy (last 3 items reverse-scored; 1 *never* – 5 *very often*). Depressive symptoms: In the past week...: I felt depressed; I could not "get going;" my sleep was restless; I felt that people disliked me; I felt lonely; people were unfriendly; I felt everything I did was an effort; I felt sad; I was happy; I enjoyed life (last 2 items reverse-scored; 0 *never/rarely* – 3 *very often*).

**Control variables**

Group-serving behavior: How often do you...?: go to events that help bring members of your racial/ethnic group closer together; spend your time with clubs, organizations or groups that focus on issues central to your racial/ethnic group; talk to others about the positive contributions your racial/ethnic group has made in society (1 *never* – 5 *very often*). Standing indicators: What is your family's annual household income? If you are not sure, please give your best estimate (1 *Under $20,000* – 7 *$150,000 or more*).

**References**

1. Soper D. Sample Size Calculator for Structural Equation Models. In: Free Statistics Calculators [Internet]. 2020 [cited 8 Jan 2020]. Available: https://www.danielsoper.com/statcalc/calculator.aspx?id=89

2. Begeny CT, Huo YJ. When identity hurts: How positive intragroup experiences can yield negative mental health implications for ethnic and sexual minorities. Eur J Soc Psychol. 2017;47: 803–817. doi:10.1002/ejsp.2292

3. Leach CW, van Zomeren M, Zebel S, Vliek MLW, Pennekamp SF, Doosje B, et al. Group-level self-definition and self-investment: A hierarchical (multicomponent) model of in-group identification. J Pers Soc Psychol. 2008;95: 144–165. doi:10.1037/0022-3514.95.1.144

4. Lachman ME, Weaver SL. The sense of control as a moderator of social class differences in health and well-being. J Pers Soc Psychol. 1998;74: 763–773. doi:10.1037/0022-3514.74.3.763

5. Prenda KM, Lachman ME. Planning for the future: A life management strategy for increasing control and life satisfaction in adulthood. Psychol Aging. 2001;16: 206–216. doi:10.1037/0882-7974.16.2.206

6. Spielberger CD. Manual for the State-Trait Anxiety Inventory STAI (form Y). Palo Alto, CA: Mind Garden; 1983.

7. Kohout FJ, Berkman LF, Evans DA, Cornoni-Huntley J. Two Shorter Forms of the CES-D Depression Symptoms Index. J Aging Health. 1993;5: 179–193. doi:10.1177/089826439300500202

8. Wills TA. Downward comparison principles in social psychology. Psychol Bull. 1981;90: 245–271. doi:10.1037/0033-2909.90.2.245

9. Bentler PM. EQS 6 Structural Equations Program Manual. Encino, CA: Multivariate Software Inc.; 2006.

10. Satorra A, Bentler PM. Model conditions for asymptotic robustness in the analysis of linear relations. Comput Stat Data Anal. 1990;10: 235–249. doi:10.1016/0167-9473(90)90004-2
